# Oral Health Interventions for Culturally and Linguistically Diverse Children and Caregivers: A RE‐AIM Systematic Review of Implementation Gaps

**DOI:** 10.1111/cdoe.70090

**Published:** 2026-07-16

**Authors:** I. Awadh, D. Ha, R. Boyd, A. Khan, L. Ball, M. Nisar, S. Zafar

**Affiliations:** ^1^ School of Dentistry, Faculty of Health, Medicine and Behavioural Sciences The University of Queensland Herston QLD Australia; ^2^ Centre for Extracellular Vesicle Nanomedicine, Faculty of Health, Medicine and Behavioural Sciences The University of Queensland South Brisbane QLD Australia; ^3^ Centre for Hearing Research, Faculty of Health, Medicine and Behavioural Sciences The University of Queensland St Lucia QLD Australia; ^4^ Centre for Community Health and Wellbeing The University of Queensland Springfield QLD Australia

**Keywords:** child, cultural competency, emigrants and immigrants, health services accessibility, healthcare disparities, oral health, systematic review

## Abstract

**Objectives:**

To characterise implementation reporting completeness of oral health interventions for culturally and linguistically diverse (CALD) children and caregivers using the RE‐AIM framework (Reach, Effectiveness, Adoption, Implementation, Maintenance).

**Methods:**

Six databases (PubMed, Embase, CINAHL, Scopus, Web of Science, Cochrane Library) were searched for studies published 2010–2025 (PROSPERO CRD42024531750; PRISMA 2020). Eligible studies evaluated oral health interventions for CALD children aged 0–18 years from immigrant, refugee, or asylum‐seeking backgrounds. Risk of bias was assessed with RoB 2, MMAT, and ROBINS‐I. A 22‐item RE‐AIM coding tool assessed reporting completeness across five domains, categorised as low (< 60%), moderate (60%–69.9%), or high (≥ 70%).

**Results:**

Eleven studies were included (USA *n* = 9, UK *n* = 1, Australia *n* = 1; samples 36–1207). Two of three RCTs were rated High risk (RoB 2), all six non‐randomised studies Serious risk (ROBINS‐I), and two mixed‐methods studies Low and Moderate (MMAT). Reporting completeness was predominantly low (72.7% of studies). Reach (84.1%) and Adoption (86.4%) were well‐documented; Effectiveness (32.3%) showed the largest gap. Clinical outcomes (dmft/DMFT) were assessed in 36.4% of studies; cost was not reported in any study. Follow‐up of 18 months or longer was limited to 18.2%, and attrition (5.6%–47%) in refugee populations reflected structural mobility rather than intervention failure.

**Conclusions:**

Reporting completeness was substantially incomplete for Effectiveness, cost, and long‐term follow‐up, though several gaps reflect structural constraints in CALD settings rather than reporting failures alone. Future studies should prospectively embed validated reporting checklists that capture intervention structure and implementation, select outcomes matched to the phase of evidence (proximal in early‐phase, clinical in adequately powered trials), and design retention strategies suited to mobile and recently resettled populations.

## 1 Introduction

Despite the existence of universal healthcare programs in many countries, disparities in healthcare access among Culturally and Linguistically Diverse (CALD) populations remain well‐documented [1]. The CALD population is defined as “people born in non‐English‐speaking countries and/or those who do not speak English at home” [2]. Government and non‐government organisations in several countries have adopted strategies, such as developing ethno‐specific services to address the unique needs of CALD clients [3]. The disparities in health outcomes extend to oral health, which is recognised by the World Health Organisation (WHO) as essential to overall well‐being [4]. Early childhood caries (ECC), though preventable, affects almost half of children worldwide [5]. If untreated, ECC can lead to pain, infection, difficulty eating and sleeping, impaired growth, and learning difficulties. CALD children face higher ECC rates and limited dental care access [6] due to barriers such as language, cost, psychosocial stress, and systemic issues [7], contributing to poorer oral health outcomes compared to non‐CALD peers [8].

Preventive programs and targeted interventions can reduce oral health disparities in CALD communities [9]; however, challenges persist globally. In Canada, parental cultural beliefs shape the oral health of Asian immigrant children [10]. In the United States, Mexican migrant [11], African American [12], and South Asian American [13] children face structural and communication barriers. Non‐Western immigrant children in Scandinavian countries [14], refugee and asylum‐seeker children in the United Kingdom [15], and immigrant children in Australia [16] similarly experience poorer oral health outcomes than majority populations.

Despite these efforts, oral health disparities among CALD children persist due to multiple interacting factors, including limited parental oral health knowledge, suboptimal brushing and dietary practices, and structural barriers to accessing preventive care [17, 18]. Interventions including home‐based brushing programmes, school‐based programmes, and culturally tailored education have shown promise in improving knowledge and behavioural outcomes [19, 20]. However, translating effective interventions into routine practice depends on more than efficacy. It also requires population reach, organisational adoption, delivery fidelity, cost, and sustainability, yet existing studies report these inconsistently [21]. A recent systematic review and meta‐analysis showed that parental oral health interventions in CALD populations have positive short‐term effects on knowledge and attitudes [22]. However, this review did not use a structured implementation science framework, pooled effectiveness results across different delivery methods without separating implementation dimensions, and did not explore the structural reasons (such as short funding cycles, participant mobility, or missing cost data) that recur across the evidence base. The field therefore lacks a synthesis of where, in implementation terms, the translation gap lies.

The RE‐AIM framework (Reach, Effectiveness, Adoption, Implementation, Maintenance) extends beyond efficacy reviews by evaluating both intervention effectiveness and real‐world implementation [23]. RE‐AIM is particularly suited to CALD oral health research for four reasons. First, its Reach dimension makes participation rates and sample representativeness explicit, which is critical where structural exclusion, including language access, undocumented status, and residential mobility, shapes who is reached. Second, Adoption captures whether interventions are taken up by the services and providers most likely to serve CALD families, rather than only by research teams. Third, Implementation surfaces the additional resourcing that CALD interventions require, including translation, interpretation, community liaison, fidelity, and cost. Fourth, Maintenance addresses whether effects and delivery persist beyond short funding cycles, a known weakness in community‐based CALD research. RE‐AIM has been applied in oral health settings. Oldroyd et al. [24] used it to evaluate a priority populations program in Melbourne, and Böhm da Costa et al. [25] applied it to a teledentistry system in Brazilian public health services. Neither addressed CALD children specifically, and neither synthesised reporting across multiple studies. No systematic review has applied RE‐AIM to oral health interventions targeting CALD children. As a result, the field cannot currently distinguish whether documented disparities persist because effective interventions do not reach CALD families, because they reach them but are not adopted by services, or because they are adopted but not sustained. These are three distinct problems requiring three distinct solutions.

This systematic review applies the RE‐AIM framework to oral health interventions for CALD children (0–18 years) and their caregivers to characterise implementation reporting completeness across the five domains and to conduct a formal risk‐of‐bias assessment of the included evidence. The review addresses three complementary questions: how interventions were implemented and adapted for CALD populations; for whom they were designed and delivered; and with what degree of methodological rigour.

## 2 Methods

This systematic review is reported according to the Preferred Reporting Items for Systematic Reviews and Meta‐Analyses (PRISMA) 2020 statement [26] (File S1). The review protocol was prospectively registered in the International Prospective Register of Systematic Reviews (PROSPERO; protocol number: CRD42024531750) [27]. The research question guiding this review was: “To what extent do oral health interventions for CALD children and caregivers report implementation dimensions across the five RE‐AIM domains, and what is the methodological quality of the underlying evidence base?” Full eligibility criteria are summarised in Table 1.

**TABLE 1:** PICOS eligibility criteria for studies of oral health interventions in CALD children and caregivers.

PICOS element	Description
P: Population details	CALD children aged 0–18 years and their parents/caregivers
I: Intervention details	Oral health interventions including preventive programs, educational interventions, community‐based programs, and culturally tailored initiatives
C: Comparison	Standard care, no intervention, or non‐culturally adapted oral health programs
O: Outcomes	Any oral health outcome reported by the included studies (clinical and non‐clinical), subsequently mapped onto the five RE‐AIM framework domains (Reach, Effectiveness, Adoption, Implementation, Maintenance)
S: Study design	RCTs, quasi‐experimental, pre‐post, cohort, and mixed‐methods studies
Exclusion criteria	Non‐CALD populations; adults only; settings outside English‐speaking host countries; no oral health intervention; no oral health outcome reported; conference abstracts, reviews, book chapters, editorials, and protocols

### 2.1 Searches

An electronic search across six databases (Web of Science, PubMed, CINAHL, Embase, Scopus, and the Cochrane Library) was conducted on 1 September 2024 and updated on 1 December 2025 for articles published between 1 January 2010 and 1 December 2025. The 2010 start date was chosen to capture 15 years of contemporary research while ensuring a manageable scope, and to coincide with increased global recognition of CALD health disparities and the emergence of policy initiatives on culturally responsive healthcare delivery. The search strategy combined MeSH terms and keywords for four concept blocks: (1) oral health and dental care; (2) CALD populations (immigrants, refugees, asylum‐seekers, ethnic minorities); (3) children and adolescents; and (4) interventions or programs. Full search strategies for each database are provided in File S2. Records were managed using EndNote and screened using Covidence.

Two reviewers (IA and SZ) independently screened the retrieved records in Covidence (Veritas Health Innovation, Melbourne, Australia). A third reviewer (LB) screened a subset of records to support conflict resolution. Titles and abstracts were initially examined to identify papers for full‐text review. Full texts were retrieved when abstracts provided insufficient detail to determine eligibility. Disagreements were resolved through discussion. Formal inter‐rater agreement statistics were not calculated.

#### 2.1.1 Inclusion Criteria

Eligible studies were peer‐reviewed and published in English, defined CALD by immigrant, refugee, or asylum‐seeking background in English‐speaking host countries, and reported measurable clinical or non‐clinical outcomes. Full eligibility criteria are detailed in Table 1.

### 2.2 Data Extraction

Two reviewers (IA and SZ) independently extracted data from each included study using a structured extraction form. Extracted items are presented in Table 2.

**TABLE 2:** Characteristics of the 11 included studies of oral health interventions in CALD children and caregivers.

Author (year), country	Sample size	Population characteristics	Study design	Intervention details	Duration/Follow up	Comparison	Outcome(s)	Limitation
Lumsden et al. (2024) USA [28]	*n* = 1207 children enrolled; *n* = 1124 preintervention data; *n* = 1130 postintervention data	Children Age (range: 24–72 months); (52.7%) Female; (88%) cavity history past 12 months; (99%) Medicaid Parents: Mean age 33 years; (88%) Hispanic, (95%) female; (62%) unemployed; (80%) ≤ high school education	Pre‐post study design	Delivered by community health workers across home, community, and clinic settings, the year‐long MSB program used a bilingual iPad app to provide education, risk assessment, goal‐setting, action planning, and follow‐up, with social service referrals and quarterly oral‐hygiene supplies	1‐year intervention; baseline visit, follow‐up visits, final visit at ~1 year	Within‐group comparison (no control group)	Therapeutic toothbrushing nearly doubled (OR = 1.79); decreased bottle/sippy use, nighttime breastfeeding, utensil sharing; increased meals/snacks at table (OR = 1.57–1.80); reduced toothbrushing/dietary barriers (all *p* < 0.001)	No control group; limited generalizability; no control for external dental interventions/messaging; possible social desirability bias; evaluation instrument limitations; clinical outcomes not assessed, unknown if behavioural changes yield clinically relevant caries effects
Alrashdi M et al. (2021B) USA [29]	Clinical oral health assessment *n* = 7 Oral‐health‐related quality of life *n* = 55	Recent refugee caregiver with at least one child < 12 years, lived in the U.S. for < 1 year; Diverse origins; low income (73%) < $2000/month Female (74%) Completed high school (51%)	RCT Baseline survey T1 & post‐educational session survey T2	Two of 1 h culturally tailored educational session for parents/caregivers on oral hygiene, fluoride, nutrition, and dental care access. Translated materials, supported by interpreters from similar cultural backgrounds	Two of 1 h sessions Follow up at 3 months and 6 months post‐intervention	Within‐group comparison (no control group)	No significant difference in DMFT/dmft scores between groups (*p* > 0.05) Both groups showed increased caries over time (*p* < 0.05) No improvement in oral‐health‐related quality of life (MOHRQoL‐P scores)	Small sample size (limited power) Caries diagnosed clinically without radiographs High dropout rate (28%) DMFT/dmft index may not capture all caries aspects (e.g., PUFA/ICDAS) Self‐reported quality of life data (parental bias)
Alrashdi et al. (2021a) USA [30]	*n* = 55 participants (for 80% power and 0.05 significance level) Over 60 recruited to account for potential attrition	Recent refugee with at least one child ≤ 12 years. Diverse nationality & languages; *F* = (74%); (51%) completed high school; (71%) monthly income ≤ $2000; (Center of Refugees' Services)	T1: RCT Baseline Survey, T2: Post‐education Survey; T3: After 3 months; T4: After 6	Culturally competent OH education sessions delivered by trained staff from the community (interpreters & bilingual providers translated OH materials; Visual demonstrations, discussions, and oral health education)	Two 1‐h sessions Follow up: Immediately; 3 months for intervention group only; 6 months for both groups	Control group: Completed two evaluations only, with no intervention Control	No statistically significant improvements in knowledge, attitudes & behaviours of parents before and after the oral health education program, in both the intervention and control groups	Low statistical power due to small sample size Study designed to detect large effects, possibly missing small but meaningful changes Potential cross‐contamination between groups (participants' social connections & shared location)
Yuan et al. (2019) UK [31]	*n* = 36 Intervention; *n* = 18 Control	Chinese; undocumented migrant mothers with infants under 8 weeks old Low English proficiency education; Low socio‐economic	Quasi‐experimental Non‐randomised, control Group pre‐intervention survey	A Chinese community health worker provided 5 culturally tailored home visits/telephone support on OH using leaflets‐distribution of oral care pack and feeding cup	12 months (Intervention timeline: 8 weeks, 4, 6, 9 and 12 months; Follow up at 8 weeks, 6 and 12 months)	Control group (no intervention) Test group: intervention group	Test group showed significant knowledge improvement (100% vs. 25% at 12 months) More participants brushed their baby's teeth (94% vs. 24%) and practiced sugar control Toothbrushing rates were higher (94% vs. 24%) No significant change was observed in maternal caries status	Small sample size; Non‐randomised design (risk of selection bias); Self‐reported behaviours (social desirability bias); Control group sought advice from researcher; Short follow‐up for clinical outcomes (D3MFT)
Ponce‐Gonzalez et al. (2019) USA [32]	*n* = 311 Participants in 12 workshops *n* = 278 Completed both surveys	Migrant farmworkers Spanish‐speaking Female (72%) Aged 25–49 years Varied education levels	Pre‐post study design	Trained community health workers led sessions; Didactic & interactive components included Spanish language	2 h program No follow‐up	Within‐group comparison (no control group)	Statistically significant increases in oral health knowledge; Correct answer percentages increased from (7%) to (27%)	No comparison group; Inconsistencies in Data Collection (surveys administered by lay leaders); Self‐reported, social desirability bias; Limited generalisability
Lumsden et al. (2019) USA [33]	*n* = 113 parent/child dyads approached *n* = 108 Participated *n* = 79 Completed follow‐up *n* = 29 Lost to follow‐up	Low‐income; Hispanic population; Children aged 2–6 years; (72.2%) Spanish‐speaking; (75.9%) foreign‐born; (98.2%) Medicaid beneficiaries	Pre‐post study design	Four trained bilingual investigators (health professionals/researchers) using the iPad app in the clinic. MSB: Bilingual iPad‐based intervention providing ECC risk assessment, targeted education, goal‐setting, and individualised behaviour change planning	Single intervention session (15–20 min) delivered during or just after a dental visit; 1‐month post‐intervention follow‐up	Within‐group comparison (no control group)	Acceptability/Feasibility: (95.6%) participation; (100%) completion; Behavioural Impact (1‐month): (96.2%) recalled intervention; (63.3%) recalled goal; (60.8%) stated goal correctly; (79.7%) took behaviour change action. Top Goals: (32.9%) Limit sweet foods/snacks; (27.8%) limit/dilute sweet beverages; (10.1%) visit dentist regularly; (8.9%) increase water	Limited Generalisability; Small, demographically homogenous clinic sample; Single exposure with only telephone follow‐up; Inability to separate program effects from usual care; Absence of post‐intervention clinical outcome data
Batliner et al. (2018) USA [34]	*n* = 579 mother newborn dyads, *n* = 290 (Intervention), *n* = 289 (Control) (88%) completed ≥ 3 of 4 MI sessions	American Indian mother/primary caregiver (15–65 years), of a newborn child (up to 3 months of age), enrolled at several locations, (e.g., local community events)	RCT	MI delivered by trained local interventionists (2‐day training, culturally adapted script); Mothers selected 2 of 8 oral health topics per visit (e.g., dental visits, sippy cup use, fluoride, brushing) MI compensation: $25 per visit; ECS (both groups): brochures, toothbrushes, and toothpaste for all family members	Four visits of 45 to 60 min at childbirth, 6, 12, and 18 months Follow‐up at 12, 24, and 36 months	Control group, ECS comparisons at each time point and across all follow‐up assessments	No baseline differences; dmfs increased both groups; intervention group lower at 36 months, not significant (*p* = 0.70); Caries prevalence rose (7%–9% to 55%–56%) by year 3 (*p* = 0.67). Active decay similar (*p* = 0.91). OH knowledge higher intervention group 12, 24 months (*p* = 0.03), not 36 months. OH behaviours declined (*p* = 0.86)	No significant intervention effects were found, despite a strong study design; Differences in trial design (e.g., control group, child age, location, setting) may have influenced outcomes; Limited generalizability
Northridge et al. (2017) USA [35]	*n* = 126 One group	Sikh‐American Punjabi speaking Mean age 49 years Caregivers; (55%) Females (33%) less than high school	Mixed‐methods (quantitative retrospective pre‐post survey + qualitative focus groups)	Sikh community health worker Culturally tailored OH workshops (interactive, OH demonstration, care access and dental insurance information)	Fortnight over 2 years for 30–75 min Survey conducted 1 month after the final workshop session No follow up	Within‐group comparison (no control group)	Behavioural: improved brushing and flossing Service access: 81.0% of 58 uninsured participants obtained dental insurance via the program; 92.6% linked to a local dental provider; Confidence: asking oral health questions rose from 4.8% to 62%; Acceptability: 95% rated Punjabi‐language workshops satisfactory	Self‐reported data (bias) Short follow‐up duration; Challenges in recruiting men; Limited generalisability (specific to Sikh immigrants); No control group for comparison
Hoeft et al. (2016) USA [36]	*n* = 105 baseline *n* = 95 post‐test1 (PT1) *n* = 79 post‐test 2 (PT2)	Spanish‐speaking parents with children aged 0–5 years Caregivers: (77%) mothers; 87% born in Mexico; ≤ 6 years of education (33%); Children: (42%) US‐born; Low‐income	Pre‐post study design	Phase 1: Four people with childcare experience as health educator's curriculum, lesson format, preferred activities& acceptability Phase 2: four‐sessions (OH knowledge & behaviour changes) (Spanish & English)	1‐month intervention, Four classes. (2 h each) Follow up Immediately & 3 months after intervention	Within‐group comparison (no control group)	Knowledge about OH: improved Bushing: BL (13%) PT1 (44%) PT2 (66%) Fluoridated toothpaste use: 86%–96% BL (57%) PT1 (67%); PT2 (95%) Parents check the child's teeth: Sweet drinks once a day or less: BL (33%); PT1 (77%); PT2 (63%) Eat sweet foods less than every day; Not: significant	No control group; Self‐reported data (risk of bias); Short follow‐up; Sample size based on budget constraints; Attrition (26% loss at 3‐month follow‐up)
Gibbs et al. (2015) Australia [37]	Baseline *n* = 521 Families *n* = 692 children Follow‐up *n* = 275 families *n* = 365 children	Families with 1–4‐year‐old children from Iraqi, Lebanese, or Pakistani migrant backgrounds; Male (19%); Female (81%)	Mixed method; Exploratory trial longitudinal evaluative; Non‐random allocation to intervention/comparison groups	Peer‐Led Community OH education sessions (two 3‐h sessions) over 2–3 weeks and a site visit to local dental services. Included oral health packs, plaque‐disclosing & cultural competence review to improve service access. Follow‐up health messages. 1 message per month for 4 months	Education sessions (two 3‐h sessions over 2–3 weeks) Health messages (4 months) Follow‐up at 18 months	Comparison group: Families from the same migrant backgrounds but residing outside the intervention area	Intervention group: (56%) less debris, (66%) less gingival inflammation, 2.65 times more brushing instruction reported; Twice‐daily brushing increased (19%) (intervention) vs. (11%) (comparison); No significant differences in caries experience, dental visits, or sugar intake	High attrition (47%) loss to follow‐up; Non‐random allocation to groups; Potential examiner bias; Limited generalisability as only three migrant groups; Self‐reported: subject to bias
Chi et al. (2014) USA [38]	*n* = 200 Completed surveys, *n* = 138 Korean surveys, *n* = 62 English surveys	Korean American caregivers of children under six; (73%) Female; (84.5%) College‐educated; (69%) Household incomes over $45K; (69%) preferring Korean; (31%) preferring English for communication	Prospective study with 2 phases: Phase 1: Flipchart development via focus groups Phase 2: Pre‐/post‐intervention survey	Phase 1: four focus, community‐based, 8 Korean American caregivers interviewed for 1 h to develop flipchart; Phase 2: one undergraduate & 2 s‐year dental students trained to deliver a single‐session 1‐on‐1 of 5 min, verbal education using bilingual Culturally tailored flipchart (Korean/English)	5 min No long‐term follow‐up	Within‐group comparison (no control group)	Primary Outcomes Knowledge: Significant improvements in fluoride safety, dental visit frequency, cavity aetiology, age of first dental visit; Self‐efficacy: Improved confidence in maintaining child's oral health. Secondary Outcomes: Cultural acceptability of flipchart	The flipchart covered multiple OH behaviours make it unclear which specific behaviours are most amenable to change; Short‐Term Focus; No Power Calculation: The study did not perform a priori sample size calculations

Abbreviations: BL, Base line; ECS, Enhanced Community Services; ICDAS, International Caries Detection and Assessment System; MI, Motivational Intervention; Mins, Minutes; MOHRQoL, Michigan Oral‐Health‐Related Quality of Life Scale‐Parent Version; MSB, MySmileBuddy; OH, Oral hygiene; PR, Participation Rate; PT1, Post test one; PT2, Post test two; PUFA, Pulp Ulceration Fistula Abscess Index.

### 2.3 Data Extraction Tool

Several implementation science frameworks were considered for this review, including Proctor's Implementation Outcomes Framework [39] and the Consolidated Framework for Implementation Research (CFIR) [40]. The RE‐AIM framework was selected as the primary data extraction tool because it uniquely combines assessment of intervention effectiveness with critical implementation dimensions including Reach, Adoption, Implementation (including fidelity), and Maintenance [41]. Unlike Proctor's framework, which focuses primarily on implementation success indicators, RE‐AIM explicitly addresses population‐level impact through its Reach dimension, capturing participation rates and representativeness of those who engage with interventions [42]. This is particularly valuable when assessing oral health interventions for CALD populations, where equitable access and participant engagement strongly influence outcomes. Additionally, RE‐AIM has established methodological precedent for use in systematic reviews to retrospectively assess reporting completeness of intervention studies [23, 43]. A 22‐item coding template (Table 3) was adapted from previously validated RE‐AIM tools [23] with modifications tailored to oral health interventions, aligned with recommendations of Holtrop et al. [43]. These adaptations ensured indicators remained clear, relevant, and applicable across diverse study designs and settings [44].

**TABLE 3:** Adapted RE‐AIM framework dimensions with indicators.

Reach	The reach of the intervention among diverse cultural groups
Participation rate (PR)	The percentage of eligible individuals who enrolled in the study, calculated as (number of participants ÷ number of eligible or invited individuals) × 100
Target Population (TP)	Which CALD groups are being reached? (e.g., language spoken, ethnicity, immigration status)
Demographic characteristics (DC)	Reported age, gender, or socio‐economic status (e.g., age 0–18, CALD background)
Recruitment methods (RMs)	Describes how participants are recruited to reach the intended population
Effectiveness	The impact of the intervention on key outcomes, including clinical and non‐clinical outcome
Clinical outcome	Measures related to oral health status, such as caries prevalence (DMFT/dmft index), Plaque Index, and Gingival Index
DMFT/dmft Index	The dmft and DMFT score counts the number of teeth that are decayed (d), missing due to caries (m) or filled because of caries (f); ‘dmft’ refers to primary teeth, ‘DMFT’ refers to permanent teeth. When a person has a dmft or DMFT score that is greater than zero, this is known as having caries experience
Plaque Index	Clinical plaque indices are used to evaluate the level and rate of plaque formation on tooth surfaces, and to test the efficacy of oral care products for removal and prevention of plaque deposits from these surfaces
Gingival Index (GI)	A tool for scoring gingival inflammation, combining visual and bleeding criteria
Non‐clinical outcome	Measures related to Caregivers' KAP, Dental care access and utilisation, The oral‐health‐related quality of life (OHRQoL)
Caregivers' KAP:	The KAP model is a structured, standardised questionnaire completed by a target population that can quantify and analyse what is known (knowledge), believed (attitudes), and done (practices) regarding a topic of interest
Knowledge (K)	Improved oral health knowledge (factors causing oral health problems, and tooth decay); Caregiver knowledge of fluoride benefits, sealant benefits; Awareness of subsidised clinics; Knowledge of Brushing Techniques
Attitudes (A)	Dentist visit (increase visit to dentist); Sugar intake (reduce sugar intake); Satisfaction with intervention
Behaviours (B)	Practice oral hygiene (floss once/day, brush twice/day): Assisting brushing (assist and monitor child during brushing time); Fluoridated toothpaste (use toothpaste contains fluoride): Self‐efficacy in oral health practices (Improved confidence in maintaining child's oral health)
Dental insurance (DI)	Increase dental insurance coverage
Dental checkups (DCs)	Optimal children's dental checkups
The oral‐health‐related quality of life (OHRQoL)	Patient‐reported outcome (PRO) measure that captures an individual's perception of how their oral health affects their overall well‐being and daily life
Adoption	Characteristics of settings, organisations, or individuals that are willing to adopt the intervention
Culturally adapted programs (CAP)	Intervention that are modified to align with the cultural values, beliefs, language, and practices of a specific target population
Collaborated with Community members (CE)	Training community members from same background, multilingual community workers
Bilingual Approach (BA)	Availability of interpreter service to participant native language throw out intervention
Translated materials (TMs)	Translated materials were provided in participants' native languages during educational sessions, in surveys, and in educational leaflets
Implementation	The evaluation of how well the intervention was delivered in practice. It includes the duration and frequency, ongoing delivery costs, and whether the intervention was delivered as intended (fidelity)
Duration (D)	The duration of the intervention
Frequency (F)	The frequency of the intervention
Cost (C)	The cost of the intervention
Fidelity (FD)	Whether the intervention was delivered as intended
Maintenance	Evaluates the long‐term use of the intervention, often assessed through follow‐up periods

The 22 indicators were distributed across the five domains: Reach (4), Effectiveness (9, comprising clinical and non‐clinical outcomes), Adoption (4), Implementation (4), and Maintenance (1). Full definitions, abbreviations, and operationalisation are provided in Table 3 [42].

Each indicator was scored as 1 (measured and reported in the study, regardless of effect direction or statistical significance) or 0 (not measured or not reported). For the participation rate indicator specifically, no study reported the rate directly; studies were scored positive (=1) when the rate was calculable by the reviewers from the numerator (participants enrolled) and denominator (eligible individuals approached or invited) provided in the study. Overall reporting completeness was calculated as the sum of positive indicators out of 22, expressed as a percentage. Domain‐level reporting was calculated as the percentage of indicators reported within each dimension. Studies were classified as low (< 60%), moderate (60%–69.9%), or high (≥ 70%) reporting completeness; thresholds were chosen a priori to provide a balanced distribution of studies across categories. Prior to independent coding, reviewers (IA and SZ) conducted a calibration exercise on three studies [29, 33, 38] to ensure consistent interpretation of RE‐AIM indicators. Discrepancies were discussed until consensus was reached, and coding definitions were refined accordingly. Two reviewers (IA and SZ) then independently assessed reporting completeness for all included studies, with disagreements resolved through discussion with a third reviewer (LB). These scores reflect what each study reported about its intervention and do not measure methodological quality, which was assessed separately using validated risk‐of‐bias tools.

### 2.4 Data Synthesis

Quantitative synthesis was not feasible owing to heterogeneity across populations, interventions, outcomes, and follow‐up durations. Findings were synthesised narratively using RE‐AIM as the organising structure. For each domain, the proportion of studies measuring and reporting each indicator was calculated; domain‐level reporting completeness was the mean of indicator percentages within that domain. Where appropriate, findings were stratified by study design and population subgroup. No meta‐analysis was conducted.

### 2.5 Risk of Bias Assessment

In addition to RE‐AIM reporting completeness, a formal risk‐of‐bias assessment was conducted for all 11 included studies using validated tools selected according to study design and following Cochrane methodological guidance. Randomised controlled trials [29, 30, 34] were assessed using the Cochrane Risk of Bias 2 (RoB 2) tool. Mixed‐methods studies [35, 37] were assessed using the Mixed Methods Appraisal Tool (MMAT). Non‐randomised studies of interventions [28, 31, 32, 33, 36, 38] were assessed using ROBINS‐I (Risk Of Bias In Non‐randomised Studies of Interventions). Two reviewers (IA and SZ) independently completed each assessment; disagreements were resolved through discussion with a third reviewer (LB). Formal inter‐rater agreement statistics were not calculated.

## 3 Results

The database searches identified 3748 records (Web of Science *n* = 1014; PubMed *n* = 771; Scopus *n* = 749; Embase *n* = 567; Cochrane Library *n* = 340; CINAHL *n* = 307). Citation chasing and grey literature added no further records. After the removal of 1245 duplicates and 767 records flagged by automation tools as ineligible, 1736 titles and abstracts entered screening. Screeners excluded 1705 records that did not meet the inclusion criteria, leaving 31 articles for full‐text review; all were retrieved. The team excluded 20 full‐text articles: intervention not implemented and/or outcomes not measured (*n* = 12), ineligible setting (*n* = 7), and adult participants only (*n* = 1). Eleven studies entered the final narrative synthesis (Figure 1). Risk‐of‐bias ratings for each included study are presented in Figure 2, with full per‐study assessments provided in File S3 (RoB 2, three RCTs), File S4 (ROBINS‐I, six non‐randomised studies), and File S5 (MMAT, two mixed‐methods studies).

**FIGURE 1:**
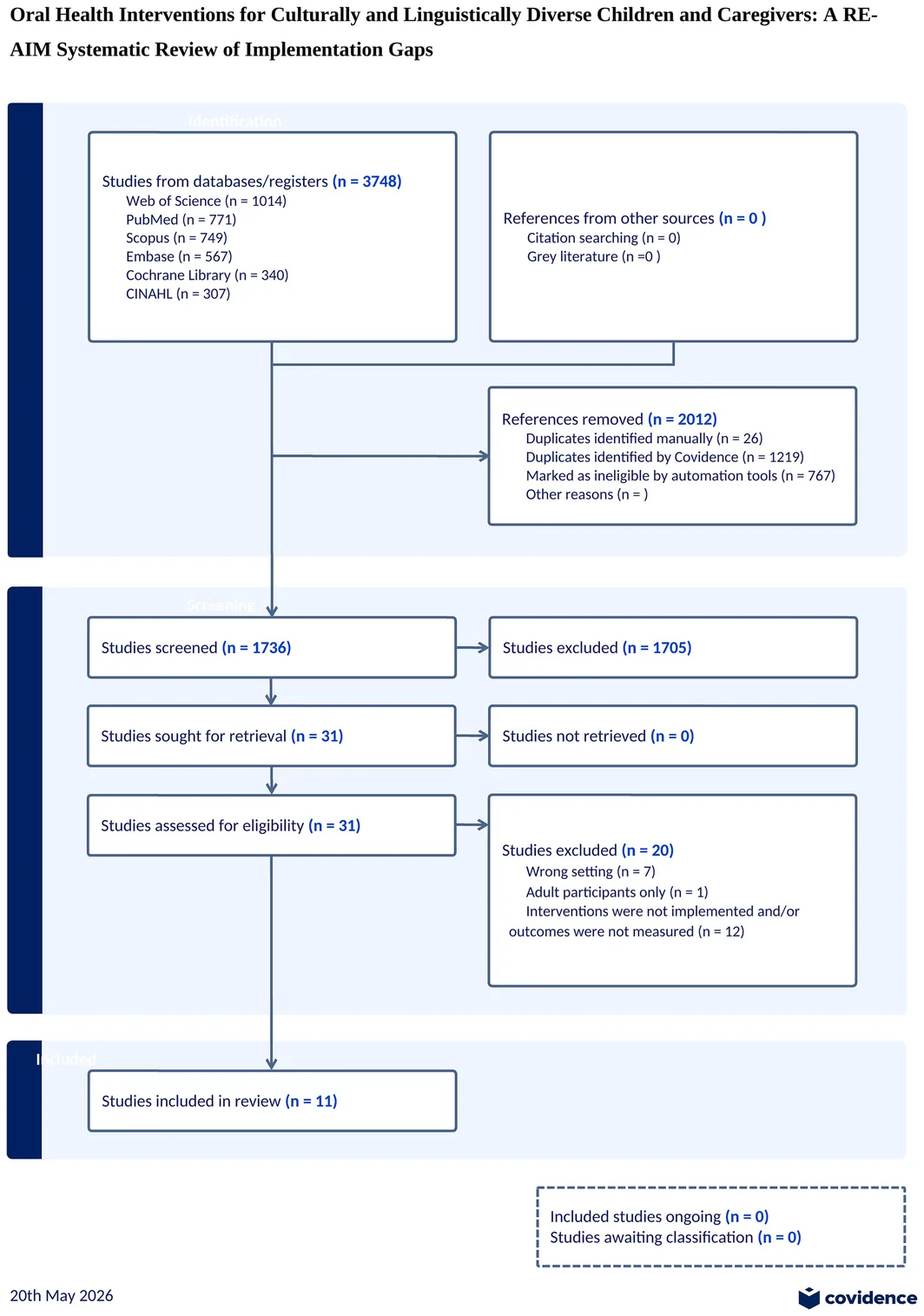
PRISMA flow chart.

**FIGURE 2:**
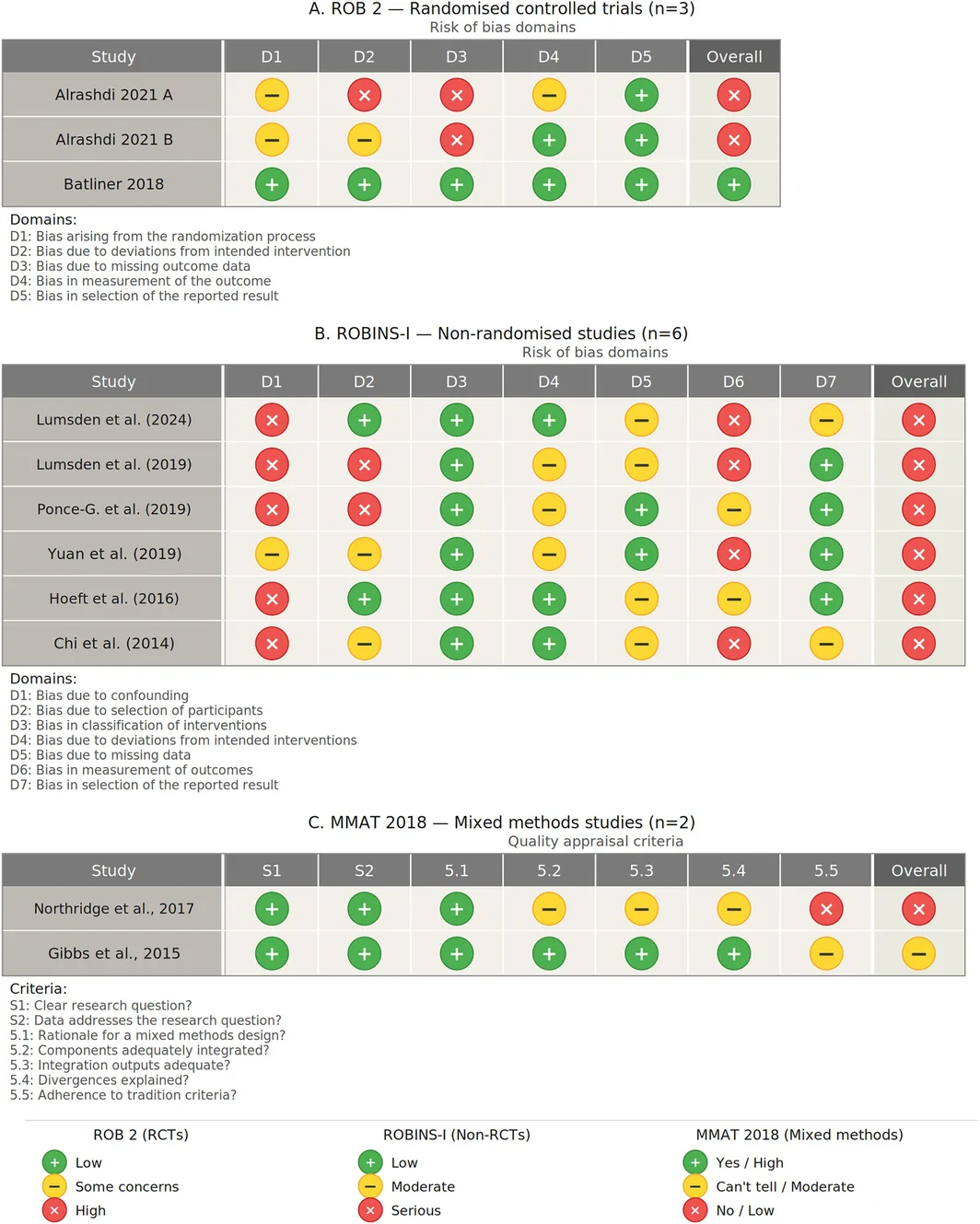
Risk‐of‐bias summary.

Overall RE‐AIM reporting completeness was categorised into three tertiles defined a priori: high (≥ 70%), moderate (60%–69.9%), and low (< 60%). These cut‐points were chosen to distinguish studies with comprehensive, partial, and limited RE‐AIM reporting. Based on this classification, low reporting completeness was found in eight studies (72.7%), moderate completeness in two (18.2%), and high completeness in one (9.1%). These scores reflect the degree to which each RE‐AIM element was documented in the study report and should not be interpreted as indicators of intervention quality or implementation effectiveness. Full details are provided in Table 4.

**TABLE 4:** Summary of RE‐AIM reporting completeness across the 11 included studies.

Author (year)	Reach				Effectiveness										Adoption			Implementation				Maintenance	Overall reporting completeness score
	PR	TP	DC	RMs	DMFT/dmft	PI	GI	K	A	B	DI	DCs	OHRQoL	CAP	CE	BA	TMs	D	F	C	FD		
Lumsden et al. (2024) [28]	Y	Y	Y	Y	X	X	X	Y	Y	Y	X	X	X	Y	Y	Y	Y	Y	Y	X	X	Y	Low
	0	1	1	1	0	0	0	0	0	1	0	0	0	1	1	1	1	1	1	0	0	1	11/22
	75%				11.1%									100%				50%				100%	50.0%
Alrashdi et al. (2021B) [29]	Y	Y	Y	Y	=	X	X	X	X	X	X	X	=	Y	Y	Y	Y	Y	Y	X	X	Y	Low
	1	1	1	1	1	0	0	0	0	0	0	0	1	1	1	1	1	1	1	0	0	1	13/22
	100%				22.2%									100%				50%				100%	59.1%
Alrashdi et al. (2021A) [30]	Y	Y	Y	Y	X	X	X	=	=	=	X	X	X	Y	Y	Y	Y	Y	Y	X	FN	Y	Moderate
	1	1	1	1	0	0	0	1	1	1	0	0	0	1	1	1	1	1	1	0	1	1	15/22
	100%				33.3%									100%				75%				100%	68.2%
Yuan et al. (2019) [31]	Y	Y	Y	Y	=	X	X	↑	↑	↑	X	X	X	Y	Y	Y	X	Y	Y	X	X	Y	Low
	0	1	1	1	1	0	0	1	1	1	0	0	0	1	1	1	0	1	1	0	0	1	13/22
	75%				44.4%									75%				50%				100%	59.1%
Ponce‐Gonzalez et al., (2019) [32]	Y	Y	Y	Y	X	X	X	↑	X	X	X	X	X	Y	Y	Y	Y	Y	Y	X	X	X	Low
	0	1	1	1	0	0	0	1	0	0	0	0	0	1	1	1	1	1	1	0	0	0	10/22
	75%				11.1%									100%				50%				0%	45.5%
Lumsden et al. (2019) [33]	Y	Y	Y	Y	X	X	X	Y	Y	Y	X	X	X	Y	X	Y	Y	Y	Y	X	X	Y	Low
	1	1	1	1	0	0	0	0	0	1	0	0	0	1	0	1	1	1	1	0	0	1	11/22
	100%				11.1%									75%				50%				100%	50.0%
T.S. Batliner et al. (2018) [34]	Y	Y	Y	Y	=	X	X	=	X	=	X	X	X	Y	Y	X	X	Y	Y	X	FY	Y	Low
	1	1	1	1	1	0	0	1	0	1	0	0	0	1	1	0	0	1	1	0	1	1	13/22
	100%				33.33%									50%				75%				100%	59.1%
Northridge et al. (2017) [35]	Y	Y	Y	Y	X	X	X	↑	↑	↑	↑	↑	X	Y	Y	Y	X	Y	Y	X	FY	X	Moderate
	0	1	1	1	0	0	0	1	1	1	1	1	0	1	1	1	0	1	1	0	1	0	14/22
	75%				55.5%									75%				75%				0%	63.6%
Hoeft KS, et al., (2016) [36]	Y	Y	Y	Y	X	X	X	↑	↑	↑	X	X	X	Y	Y	Y	Y	Y	Y	X	X	Y	Low
	0	1	1	1	0	0	0	1	1	1	0	0	0	1	1	1	1	1	1	0	0	1	13/22
	75%				33.3%									100%				50%				100%	59.1%
Gibbs et al. (2015) [37]	Y	Y	Y	Y	=	↑	↑	↑	=	↑	X	↑	X	Y	Y	Y	X	Y	Y	X	FY	Y	High
	0	1	1	1	1	1	1	1	1	1	0	1	0	1	1	1	0	1	1	0	1	1	17/22
	75%				77.7%									75%				75%				100%	77.3%
Donald L Chi, et al., (2014) [38]	Y	Y	Y	Y	X	X	X	Y	X	Y	X	X	X	Y	Y	Y	Y	Y	Y	X	FY	X	Low
	0	1	1	1	0	0	0	1	0	1	0	0	0	1	1	1	1	1	1	0	1	0	12/22
	75%				22.2%									100%				75%				0%	54.5%

Abbreviations: =, indicator mentioned and used but did not change pre‐post intervention; ↑, indicator improved after intervention; 0, indicator was not mentioned or used; 1, indicator mentioned and used, regardless of effect direction (Y, ↑, =, FY, or FN); A, Attitude; B, Behaviour; BA, Bilingual approach; C, Cost; CAP, Culturally adapted programs; CE, Collaboration with community members; D, Duration; DC, Demographic characteristics; DCs, Dental checkups; DI, Dental insurance; F, Frequency; FD, Fidelity. X, indicator not mentioned or used; FN, intervention was not delivered as intended; FY, intervention was delivered as intended; GI, Gingival Index; K, Knowledge; OHRQoL, oral‐health‐related quality of life; PI, Plaque Index; PR, Participation rate; RMs, Recruitment methods; TMs, Translated materials; TP, Target Population; Y, indicator mentioned or used.

Study designs comprised randomised controlled trials (*n* = 3) [29, 30, 34], pre‐post studies (*n* = 4) [28, 32, 33, 36], mixed‐methods studies (*n* = 2) [35, 37], one quasi‐experimental study [31], and one prospective study with pre/post‐intervention survey [38].

The included studies were conducted predominantly in the USA (*n* = 9) [28, 29, 30, 32, 33, 34, 35, 36, 38], with one study each from the UK [31] and Australia [37]. Sample sizes ranged from 36 to 1207 participants, with follow‐up durations varying from single‐session assessments to 36 months.

The included studies reflected a broad representation of CALD populations. Prominent groups included Hispanic/Spanish‐speaking caregivers, particularly from Mexican and Central American backgrounds [28, 32, 33, 36], recently resettled refugees from regions including Africa, the Middle East, and South America [29, 30], and Korean‐American caregivers [38]. Other groups included Sikh‐American Punjabi‐speaking participants [35], American Indian mothers [34], Chinese undocumented migrant mothers in the UK [31], and migrant families of Iraqi, Lebanese, and Pakistani heritage in Australia [37].

Across the included interventions (*n* = 11), the settings reflected a clear emphasis on accessibility and community integration. Most studies delivered the intervention in community‐based venues, such as cultural or religious centres, community organisations, and public events embedded within participants' own neighbourhoods [32, 34, 35, 36, 37, 38]. Two studies delivered the intervention through refugee service organisations functioning as refugee centres, providing care within familiar settlement support environments [29, 30]. One study was delivered through home visits by a community health worker [31]. One study was integrated into clinical encounters within a dental clinic setting [33]. One study adopted a flexible mixed model in which families could receive the programme at home, in community venues, or in clinic locations according to their preferences and practical constraints [36].

Intervention content and delivery methods were heterogeneous but fell into three main categories: oral health education, motivational interviewing, and structured workshops. Ten studies used oral health education, targeting caregivers' knowledge and behaviours around hygiene, diet, and dental service use, and delivered via home visits, brief one‐to‐one sessions, and multi‐session programmes in community or refugee venues [28, 29, 30, 31, 32, 33, 35, 36, 37, 38]. One study used motivational interviewing [34], delivered by trained local community members. Six studies used group‐based, interactive educational workshops, led by peer educators or community health workers in trusted community or refugee settings [29, 30, 32, 35, 36, 37].

Some interventions incorporated preventive components such as oral health packs or supplies, including toothbrushes and toothpaste [28, 31, 34, 37], alongside facilitated links to services or referrals where relevant [28, 35, 37]. Formats ranged from brief, single‐session interventions of around 5–20 min [33, 38] to structured multi‐session workshops, for example multiple classes over several weeks [36], and longer programmes delivered over 12 months or more [28, 31, 34], with follow‐up periods varying from immediate post‐intervention assessment to 36 months. One study used a fortnightly delivery schedule spanning 2 years [35]. Hybrid models combining face‐to‐face delivery with telephone support were also used [31]. A summary of study characteristics is presented in Table 2.

Female caregivers predominated across the included studies, with minimal participation by male caregivers. Several interventions explicitly targeted mothers or parent–child dyads [28, 29, 30, 31, 33, 34, 36], and female participation exceeded 70% in 10 of the 11 studies [28, 29, 30, 31, 32, 33, 34, 36, 37, 38]. Only one study reported a near‐balanced sex distribution, with 55% female participants [35].

### 3.1 Outcome Measures Aligned With the RE‐AIM Framework

Mean per‐study Reach score was 3.4 of 4. Indicator‐level reporting completeness was 37 of 44 (84.1%). A participation rate was calculable in four studies (36.4%) [29, 30, 33, 34], the highest being Lumsden et al. [33], which reported that 95.6% of eligible dyads approached agreed to participate. All 11 studies (100%) reported the number of participants enrolled and the final sample size [28, 29, 30, 31, 32, 33, 34, 35, 36, 37, 38], and all described the intended target population, with participants drawn from specified cultural and linguistic groups; reports captured ethnicity, language, country of origin, and immigration or refugee status [28, 29, 30, 31, 32, 33, 34, 35, 36, 37, 38]. All 11 studies (100%) described demographic characteristics, including age, gender, and socioeconomic indicators [28, 29, 30, 31, 32, 33, 34, 35, 36, 37, 38]. All 11 studies (100%) reported recruitment methods [28, 29, 30, 31, 32, 33, 34, 35, 36, 37, 38], commonly through culturally responsive channels such as community centres [30], cultural associations [35], and community events [34].

Mean per‐study Effectiveness score was 2.9 of 9. Indicator‐level reporting completeness was 32 of 99 (32.3%). Clinical outcomes were not reported as frequently as non‐clinical outcomes; caries experience (DMFT/dmft) was reported in four studies (36.4%) [29, 31, 34, 37], none of which showed statistically significant improvement after intervention. Plaque and gingival indices were each reported in only one study (9.1%) [37]. Caregiver oral health knowledge was reported in eight studies (72.7%) [30, 31, 32, 34, 35, 36, 37, 38]; of these, five (62.5%) showed statistically significant improvements [31, 32, 35, 36, 37]. Attitudes were reported in five studies (45.5%) [30, 31, 35, 36, 37]; of these, three (60.0%) demonstrated significant positive shifts [31, 35, 36]. Behavioural outcomes were reported in nine studies (81.8%) [28, 30, 31, 33, 34, 35, 36, 37, 38]; of these, four (44.4%) showed statistically significant improvements [31, 35, 36, 37]. Dental insurance was reported in one study (9.1%) [35], and oral‐health‐related quality of life in one study (9.1%) [29]. Regular dental check‐up attendance was reported in two studies (18.2%) [35, 37], with increases observed in both. Only two studies (18.2%) [34, 37] reported follow‐up at 18 months or longer, with the longest at 36 months [34].

Mean per‐study Adoption score was 3.45 of 4. Indicator‐level reporting completeness was 38 of 44 (86.4%). All 11 studies (100%) [28, 29, 30, 31, 32, 33, 34, 35, 36, 37, 38] reported some form of cultural adaptation of the intervention (CAP), aligned with the language, beliefs, and practices of the target community. Ten studies (90.9%) reported collaboration with community members from similar cultural backgrounds (CE) [28, 29, 30, 31, 32, 34, 35, 36, 37, 38]. Ten studies (90.9%) used bilingual delivery or interpreter services (BA) [28, 29, 30, 31, 32, 33, 35, 36, 37, 38]. Seven studies (63.6%) provided translated materials (TMs), such as culturally and linguistically adapted educational resources, questionnaires, or take‐home leaflets [28, 29, 30, 32, 33, 36, 38].

Mean per‐study Implementation score was 2.5 of 4. Indicator‐level reporting completeness was 27 of 44 (61.4%). Duration (D) and frequency (F) were reported in all 11 studies (100%) [28, 29, 30, 31, 32, 33, 34, 35, 36, 37, 38]. Intervention duration ranged from a single 5‐min session [38] to fortnightly workshops delivered over 2 years [35]; full per‐study detail is given in Table 2. Fidelity (FD) was reported in five studies (45.5%) [30, 34, 35, 37, 38]; of these, four (80.0%) reported that the intervention was delivered as intended [34, 35, 37, 38], and one (20.0%) reported that it was not [30]. Intervention costs (C) were not reported in any of the 11 studies (0%).

Mean per‐study Maintenance score was 0.73 of 1. Indicator‐level reporting completeness was 8 of 11 (72.7%). Long‐term sustainability or follow‐up beyond the immediate post‐intervention assessment was reported in eight studies (72.7%) [28, 29, 30, 31, 33, 34, 36, 37]. The remaining three studies (27.3%) [32, 35, 38] conducted only immediate post‐intervention assessments. Among the eight studies with follow‐up data, follow‐up duration ranged from 1 month [33] to 36 months [34]: one study at 1 month [33], five studies at 3–12 months [28, 29, 30, 31, 36], and two studies at 18 months or longer [34, 37], with the longest at 36 months [34]. Setting‐level maintenance (whether the intervention was integrated into routine service delivery) was not reported in any included study.

Attrition was reported in six of the eight studies with follow‐up data. Rates ranged from 5.6% over 12 months in Yuan [31] to approximately 47% at 18 months in Gibbs [37]. Other figures were 28% at 6 months in the Alrashdi trial [29, 30], 26.9% at 1 month in Lumsden et al. [33], and approximately 25% cumulatively over 3 months in Hoeft [36]. Batliner [34] reported retention above 80% at every planned measurement point and 88% completion of at least three of four follow‐ups through 36 months. Attrition was not clearly reported in Lumsden et al. [28].

## 4 Discussion

This systematic review applied the RE‐AIM framework to characterise implementation reporting completeness of oral health interventions for CALD children and caregivers across 11 studies from the USA, UK, and Australia. Reporting differed substantially across domains. Reach and Adoption were consistently well documented, indicating that culturally responsive recruitment and delivery are achievable in CALD settings. Effectiveness, Implementation, and Maintenance showed the most pronounced gaps, with clinical outcomes, intervention costs, and long‐term follow‐up systematically absent. These patterns reflect both documentation shortfalls and structural constraints endemic to community‐based research with underserved populations.

Most interventions engaged CALD populations through community networks, bilingual facilitators, and translated materials, consistent with evidence that culturally congruent delivery improves participation and retention [45, 46, 47]. However, recruitment through established organisations introduces a systematic selection bias: populations who are socially isolated, recently arrived, or not connected to community services, arguably those with the greatest oral health need, are least likely to be reached. Participation rates remained largely uncharacterised, leaving the representativeness of enrolled samples unclear. The absence of African and Pacific Islander communities, despite their disproportionate early childhood caries burden, is a design gap rather than a reporting omission and represents a critical equity failure.

Low Effectiveness reporting reflects two distinct problems that should not be conflated. The first is documentation: many studies did not report outcomes that were likely measured, particularly validated clinical indices. The second is design: several studies were brief community education sessions, such as Chi et al. [38] and Lumsden et al. [33], for which caregiver knowledge and behavioural intention were the appropriate primary endpoints, not caries incidence. Treating the absence of clinical outcomes in these studies as an effectiveness failure misrepresents the literature. For early‐phase CALD research, proximal behavioural and knowledge outcomes are clinically meaningful intermediate endpoints, and expecting caries‐level changes within 12 months in community‐based settings is methodologically unrealistic. Findings also varied by community context. Interventions delivered through trusted community health workers, including Yuan et al. [31] among Chinese migrant mothers in Belfast and Northridge et al. [35] among Sikh‐American families, reported gains in caregiver knowledge and dental service access. By contrast, studies of recently resettled refugees [29, 30] showed no significant improvements, a pattern likely reflecting compounding structural adversity (trauma exposure, housing instability, competing resettlement demands) that educational interventions alone cannot address. Where clinical outcomes were measured, Alrashdi [29] reported caries increases in both arms with no significant intervention effect, consistent with the limited capacity of education to alter clinical trajectories when access barriers persist.

Cultural adaptation was universal, most often through bilingual facilitators, community member collaboration, and translated materials, yet the processes underpinning adaptation were rarely evaluated with rigour. Few studies used validated cultural adaptation instruments or quantified the contribution of specific components to outcomes. The Ecological Validity Model [48], which structures cultural adaptation across eight contextual dimensions (language, persons, metaphors, content, concepts, goals, methods, and context), offers a framework for future CALD oral health research. Intervention intensity also varied widely. Brief single‐contact sessions can raise short‐term awareness, but durable behaviour change needs repeated reinforcement and consistent delivery by trained staff, particularly in CALD settings where trust‐building and navigation support take time.

Fidelity reporting varied in rigour. Three studies used systematic monitoring: audio review with the MITI 3.1.1 instrument [34], structured observation against a training manual [37], and combined self‐report with research‐staff observation [35]. One used delivery standardisation through staff training [38]. One study explicitly acknowledged the absence of fidelity assessment [30], and the remaining six did not report fidelity at all. Alrashdi [30] is an instructive exception: the authors openly acknowledged low fidelity to the motivational interviewing model and identified this as a likely reason for their null result. This explicit link between an implementation gap and a null finding is what implementation‐focused reporting should surface; its absence elsewhere means null findings across the literature cannot be confidently attributed to intervention design, delivery, or both.

No study reported intervention costs, a critical evidence gap given that translation, interpretation, and community liaison add substantial resource requirements that affect feasibility and scalability in CALD contexts [49, 50]. Refugee‐focused studies [29, 30] documented contextual delivery challenges, including restricted access to dental treatment alongside the educational intervention and difficulty maintaining contact with highly mobile families. Such constraints should be reported as part of implementation evaluation rather than treated as incidental limitations.

Long‐term and setting‐level Maintenance were both underreported. Caries detection requires standardised methods capable of capturing lesion change over time [51], and caries trials are typically designed with follow‐up of 24 months or longer; only one study reported follow‐up beyond 18 months [34]. The absence of setting‐level Maintenance reporting likely reflects short funding cycles and the absence of established adoption pathways from research projects into routine dental services in CALD‐serving health systems.

Attrition varied widely and warrants contextual interpretation, not uniform classification as a methodological shortcoming. In studies involving recently resettled refugees [28, 29, 30, 33], elevated attrition more plausibly reflects structural instability and competing resettlement demands than disengagement from the intervention. Under these conditions, attrition is itself an informative outcome and should be reported accordingly. Batliner [34] and Yuan [31] illustrate that strong retention is achievable when delivery is embedded in trusted relationships, through participant incentives and community‐embedded delivery [34], or through community health worker home visits and telephone contact [31]. Future studies should adopt flexible contact protocols, community liaison infrastructure, and intention‐to‐treat analyses as standard design components.

Several limitations require acknowledgement. The risk‐of‐bias assessment rated all six non‐randomised studies as Serious under ROBINS‐I and two of three RCTs as High under RoB 2, so confounding and selection bias are likely to overestimate intervention effects. Reporting completeness and methodological quality are distinct constructs, captured by RE‐AIM scores and risk‐of‐bias assessments respectively. Restricted RE‐AIM reporting further constrained narrative synthesis, particularly for Effectiveness and Implementation. Geographic concentration in the USA limits transferability, and the English‐language restriction may have excluded relevant evidence from non‐English‐speaking high‐income settings. Inter‐rater agreement statistics were not formally calculated; coding was resolved by discussion between authors, which may have introduced consensus bias.

### 4.1 Implications for Future CALD Oral Health Intervention Research

These findings translate into a set of design and reporting priorities for future CALD oral health intervention research. Researchers should apply an implementation assessment framework at the design stage and pre‐specify reporting criteria: RE‐AIM, CFIR, and Proctor's Implementation Outcomes Framework are options. Investigators should match outcomes to study phase: proximal endpoints in early‐phase studies, clinical outcomes in trials with follow‐up of 24 months or longer. Journals and funders should require cost and fidelity reporting as standard, and systematic reviewers should score reporting completeness and methodological quality separately. Research teams should co‐design interventions with community organisations through participatory methods, and trialists should test multi‐component interventions combining sustained community health worker engagement with structural navigation support. Priority research areas include African and Pacific Islander communities, comparative effectiveness across CALD subgroups facing distinct structural barriers, cost‐effectiveness analyses, and integration of oral health interventions into routine dental services.

## 5 Conclusion

The evidence base for oral health interventions in CALD populations rests on short, project‐based studies that under‐report implementation and effectiveness. Culturally responsive recruitment and delivery are feasible, but cultural adaptation cannot offset structural barriers to dental care access. CALD oral health intervention research needs prospectively designed trials that embed implementation assessment from inception, match outcomes to study phase, and report cost, fidelity, and long‐term follow‐up as standard. Funders and journals should prioritise African and Pacific Islander communities, currently absent from the evidence base despite their disproportionate caries burden.

## Author Contributions

Israa Awadh: Conceptualisation; methodology; data curation; formal analysis; investigation; writing: original draft; review and editing. Diep Ha: Supervision. Roslyn Boyd: writing – review and editing. Asaduzzaman Khan: Supervision; writing – review and editing. Lauren Ball: Methodology, supervision; writing – review and editing. Mehwish Nisar: Methodology; writing – review and editing. Sobia Zafar: Conceptualisation; methodology; data curation; project administration; supervision; writing – review and editing. All authors have read and approved the final version of the manuscript.

## Funding

The authors have nothing to report.

## Conflicts of Interest

The authors declare no conflicts of interest.

## Supporting information


**File S1:** PRISMA 2020 checklist.
**File S2:** Electronic databases searched and search strategies used in the systematic review (from 2010 up to Dec 2025).
**File S3:** Risk of bias assessments using RoB 2 for the three included randomised controlled trials.
**File S4:** The Risk Of Bias In Non‐randomized Studies – of Interventions (ROBINS‐I) assessment tool.
**File S5:** Quality appraisals using the Mixed Methods Appraisal Tool (MMAT) for the two included mixed‐methods studies.

## Data Availability

The data supporting the findings of this study are available within the article and its Supporting Information.
