# Supplementary material for: Oral Health Interventions for Culturally and Linguistically Diverse Children and Caregivers: A RE‐AIM Systematic Review of Implementation Gaps
Source: Community Dent Oral Epidemiol. 2026 Jul 16;54(5):527–45. doi: 10.1111/cdoe.70090 (PMC13669799; doi:10.1111/cdoe.70090)
Supplement: Supplementary file 1 [file CDOE-54-527-s001.zip › cdoe70090-sup-0001-Supinfo1@Supplementary_File_Captions.docx]

**Supplementary File Captions**

*Manuscript: Oral Health Interventions for Culturally and Linguistically Diverse Children and Caregivers: A RE-AIM Systematic Review of Implementation Gaps (ID 8810348)*

**Supplementary File S3.** Risk of bias assessments using RoB 2 for the three included randomised controlled trials.

**Supplementary File S5.** Quality appraisals using the Mixed Methods Appraisal Tool (MMAT) for the two included mixed-methods studies.
