# Supplementary material for: Oral Health Interventions for Culturally and Linguistically Diverse Children and Caregivers: A RE‐AIM Systematic Review of Implementation Gaps
Source: Community Dent Oral Epidemiol. 2026 Jul 16;54(5):527–45. doi: 10.1111/cdoe.70090 (PMC13669799; doi:10.1111/cdoe.70090)
Supplement: Supplementary file 1 [file CDOE-54-527-s001.zip › cdoe70090-sup-0004-Supinfo4@Supplementary_File_S4_ROBINS-I_Assessments.pdf]

The Risk Of Bias In Non-randomized Studies – of Interventions (ROBINS-I) assessment tool

(version for cohort-type studies)

Version 19 September 2016

This work is licensed under a Creative Commons Attribution-NonCommercial-NoDerivatives 4.0 International License.

ROBINS-I tool (Stage I): At protocol stage

Specify the review question

Participants	CALD caregivers (mothers/parents) of children aged 0–18 years from immigrant, refugee, or asylum-seeking backgrounds
Experimental intervention	Culturally tailored oral health education or preventive intervention
Comparator	No intervention or usual care
Outcomes	Oral health knowledge, attitudes, and behaviours; caries experience (DMFT/dmft, defs/DMFS); plaque index; gingival index; oral hygiene index; OHRQoL; fluoride varnish uptake; dental service utilisation and access to care; caries arrest or prevention rates; dental pain or infection episodes.

List the confounding domains relevant to all or most studies

Baseline oral health knowledge and attitudes; baseline caries experience (clinical status); socioeconomic status; parental education level; language proficiency; length of residence in host country; number of children; access to dental services; child age; follow-up length; incentives provided to participants.

List co-interventions that could be different between intervention groups and that could impact on outcomes

Receipt of general health education or home visiting services outside the study; access to community health workers; dental service attendance during the study period; provision of oral hygiene materials (toothbrushes, toothpaste) as part of the intervention; financial incentives per session or survey attendance; fluoride varnish application during routine dental visits.

Study ID: Chi et al. 2014 [Ref 34]

ROBINS-I tool (Stage II): For each study

Specify a target randomized trial specific to the study

Design	Individually randomized
Participants	Korean-American caregivers of preschoolers <6 yrs, Seattle, WA
Experimental intervention	Single session 5-min bilingual flipchart education delivered by trained students
Comparator	No comparator

Is your aim for this study…?

- ☐ to assess the effect of *assignment to* intervention
- ☐ to assess the effect of *starting and adhering to* intervention

Specify the outcome

Specify which outcome is being assessed for risk of bias (typically from among those earmarked for the Summary of Findings table). Specify whether this is a proposed benefit or harm of intervention.

OH knowledge and self-efficacy (proposed benefit); pre-post survey; stratified by acculturation

Specify the numerical result being assessed

In case of multiple alternative analyses being presented, specify the numeric result (e.g. RR = 1.52 (95% CI 0.83 to 2.77) and/or a reference (e.g. to a table, figure or paragraph) that uniquely defines the result being assessed.

Self-efficacy improved (p<0.0001); fluoride toothpaste safety knowledge improved (p<0.0001) — Table 2

Preliminary consideration of confounders

Complete a row for each important confounding domain (i) listed in the review protocol; and (ii) relevant to the setting of this particular study, or which the study authors identified as potentially important.

"Important" confounding domains are those for which, in the context of this study, adjustment is expected to lead to a clinically important change in the estimated effect of the intervention. "Validity" refers to whether the confounding variable or variables fully measure the domain, while "reliability" refers to the precision of the measurement (more measurement error means less reliability).

**(i) Confounding domains listed in the review protocol**				
Confounding domain	Measured variable(s)	Is there evidence that controlling for this variable was unnecessary?*	Is the confounding domain measured validly and reliably by this variable (or these variables)?	OPTIONAL: Is failure to adjust for this variable (alone) expected to favour the experimental intervention or the comparator?
Baseline OH knowledge	Pre-survey	No	Yes	No information
Acculturation level	Language preference (proxy)	No	Yes	No information
SES	Annual household income	No	Yes	No information
Education level	Self-reported	No	Yes	No information
History/maturation effects	No control group	No	No	Favour experimental

**(ii) Additional confounding domains relevant to the setting of this particular study, or which the study authors identified as important**				
Confounding domain	Measured variable(s)	Is there evidence that controlling for this variable was unnecessary?*	Is the confounding domain measured validly and reliably by this variable (or these variables)?	OPTIONAL: Is failure to adjust for this variable (alone) expected to favour the experimental intervention or the comparator?
Student deliverer variability	Not measured	No	Yes	No information

* In the context of a particular study, variables can be demonstrated not to be confounders and so not included in the analysis: (a) if they are not predictive of the outcome; (b) if they are not predictive of intervention; or (c) because adjustment makes no or minimal difference to the estimated effect of the primary parameter. Note that "no statistically significant association" is not the same as "not predictive".

Preliminary consideration of co-interventions

Complete a row for each important co-intervention (i) listed in the review protocol; and (ii) relevant to the setting of this particular study, or which the study authors identified as important.

"Important" co-interventions are those for which, in the context of this study, adjustment is expected to lead to a clinically important change in the estimated effect of the intervention.

**(i) Co-interventions listed in the review protocol**		
Co-intervention	Is there evidence that controlling for this co-intervention was unnecessary (e.g. because it was not administered)?	Is presence of this co-intervention likely to favour outcomes in the experimental intervention or the comparator
Dental attendance during study	No	No information
General health education outside study	No	No information
OH materials provided	No / flipchart, toothbrush, toothpaste given to all	Favour experimental

**(ii) Additional co-interventions relevant to the setting of this particular study, or which the study authors identified as important**		
Co-intervention	Is there evidence that controlling for this co-intervention was unnecessary (e.g. because it was not administered)?	Is presence of this co-intervention likely to favour outcomes in the experimental intervention or the comparator
Q&A session after flipchart (beyond standardised protocol)	No	Favour experimental

Risk of bias assessment

Responses underlined in green are potential markers for low risk of bias, and responses in red are potential markers for a risk of bias. Where questions relate only to sign posts to other questions, no formatting is used.

Signalling questions	Description	Response options
**Bias due to confounding**		
1.1 Is there potential for confounding of the effect of intervention in this study?<br>**If N/PN to 1.1:** the study can be considered to be at low risk of bias due to confounding and no further signalling questions need be considered	No control group; self-selected community sample; acculturation differences between subgroups uncontrolled at whole-sample level	Y
**If Y/PY to 1.1**: determine whether there is a need to assess time-varying confounding:		
1.2. Was the analysis based on splitting participants' follow up time according to intervention received?<br>**If N/PN**, answer questions relating to baseline confounding (1.4 to 1.6)<br>**If Y/PY**, go to question 1.3.	Single immediate post-intervention measurement only	N
1.3. Were intervention discontinuations or switches likely to be related to factors that are prognostic for the outcome?<br>**If N/PN**, answer questions relating to baseline confounding (1.4 to 1.6)<br>**If Y/PY**, answer questions relating to both baseline and time-varying confounding (1.7 and 1.8)		NA

**Questions relating to baseline confounding only**		
1.4. Did the authors use an appropriate analysis method that controlled for all the important confounding domains?	stratified by acculturation but no adjustment for SES, education, or other confounders	PN
1.5. **If Y/PY to 1.4**: Were confounding domains that were controlled for measured validly and reliably by the variables available in this study?	language preference as acculturation proxy is limited; other confounders unmeasured	PN
1.6. Did the authors control for any post-intervention variables that could have been affected by the intervention?	Q&A session and OH materials not controlled	N
**Questions relating to baseline and time-varying confounding**		
1.7. Did the authors use an appropriate analysis method that controlled for all the important confounding domains and for time-varying confounding?		NA
1.8. **If Y/PY to 1.7**: Were confounding domains that were controlled for measured validly and reliably by the variables available in this study?		NA
**Risk of bias judgement**		Moderate
Optional: What is the predicted direction of bias due to confounding?	better than most pre-post studies, baseline measured prospectively, acculturation stratification attempted. However, no control group and residual confounding remains.	Favours experimental

Bias in selection of participants into the study		
2.1. Was selection of participants into the study (or into the analysis) based on participant characteristics observed after the start of intervention?<br>**If N/PN to 2.1:** go to 2.4	Recruited before intervention at community venues	N
2.2. **If Y/PY to 2.1**: Were the post-intervention variables that influenced selection likely to be associated with intervention?		NA
2.3 **If Y/PY to 2.2**: Were the post-intervention variables that influenced selection likely to be influenced by the outcome or a cause of the outcome?		
2.4. Do start of follow-up and start of intervention coincide for most participants?	Pre-survey administered immediately before flipchart session	Y
2.5. **If Y/PY to 2.2 and 2.3, or N/PN to 2.4**: Were adjustment techniques used that are likely to correct for the presence of selection biases?		NA
**Risk of bias judgement**	Purposive community sample from churches, community centres, festivals — self-selected, health-interested. No control group. However recruitment setting and process clearly described.	Moderate
Optional: What is the predicted direction of bias due to selection of participants into the study?		Favours experimental / Favours comparator / Towards null /Away from null / Unpredictable

Bias in classification of interventions		
3.1 Were intervention groups clearly defined?	5-min standardised flipchart session, protocol clearly described	Y
3.2 Was the information used to define intervention groups recorded at the start of the intervention?	Pre-survey administered before intervention, protocol pre-specified	Y
3.3 Could classification of intervention status have been affected by knowledge of the outcome or risk of the outcome?	Single group, no misclassification possible	N
**Risk of bias judgement**		Low
Optional: What is the predicted direction of bias due to classification of interventions?		N/A

Bias due to deviations from intended interventions		
**If your aim for this study is to assess the effect of assignment to intervention, answer questions 4.1 and 4.2**		
4.1. Were there deviations from the intended intervention beyond what would be expected in usual practice?	Q&A session after flipchart allowed; OH materials (toothbrush, toothpaste, flipchart copy) provided as incentive	PN
4.2. **If Y/PY to 4.1**: Were these deviations from intended intervention unbalanced between groups *and* likely to have affected the outcome?	no comparator; all participants received same deviations so imbalance cannot be assessed	PN
**If your aim for this study is to assess the effect of starting and adhering to intervention, answer questions 4.3 to 4.6**		
4.3. Were important co-interventions balanced across intervention groups?		NA
4.4. Was the intervention implemented successfully for most participants?		NA
4.5. Did study participants adhere to the assigned intervention regimen?		NA
4.6. **If N/PN to 4.3, 4.4 or 4.5**: Was an appropriate analysis used to estimate the effect of starting and adhering to the intervention?		NA
**Risk of bias judgement**	Deviations were minor, applied equally to all participants, and protocol was standardised with trained deliverers.	Low
Optional: What is the predicted direction of bias due to deviations from the intended interventions?		Favours experimental / Favours comparator / Towards null /Away from null / Unpredictable

Bias due to missing data		
5.1 Were outcome data available for all, or nearly all, participants?	91.3% had usable data (200/219)	PY
5.2 Were participants excluded due to missing data on intervention status?	All 219 received the intervention in a single session (no intervention status to be missing)	N
5.3 Were participants excluded due to missing data on other variables needed for the analysis?	15 excluded for uncertainty about child's age, 4 for incomplete or unclear responses	Y
5.4 **If PN/N to 5.1, or Y/PY to 5.2 or 5.3**: Are the proportion of participants and reasons for missing data similar across interventions?	No breakdown of exclusions by language stratum	NI
5.5 **If PN/N to 5.1, or Y/PY to 5.2 or 5.3**: Is there evidence that results were robust to the presence of missing data?	No sensitivity analysis or imputation	PN
**Risk of bias judgement**	Modest loss, administrative reasons unlikely linked to outcome, but no robustness checks reported.	Moderate
Optional: What is the predicted direction of bias due to missing data?		Unpredictable

Bias in measurement of outcomes		
6.1 Could the outcome measure have been influenced by knowledge of the intervention received?	Self-report knowledge survey administered immediately after teaching; participants knew the "correct" answers	Y
6.2 Were outcome assessors aware of the intervention received by study participants?	Same students delivered intervention AND administered post-survey, no blinding	Y I
6.3 Were the methods of outcome assessment comparable across intervention groups?	Same survey instrument used pre and post (though post had only 13 of 19 items)	PY
6.4 Were any systematic errors in measurement of the outcome related to intervention received?	Systematic error likely: testing effect, social desirability, demand characteristics all push scores up post-intervention	Y
**Risk of bias judgement**	The outcome is self-reported knowledge measured minutes after teaching, by the people who just taught it. This is the weakest possible measurement design for an educational intervention.	Serious
Optional: What is the predicted direction of bias due to measurement of outcomes?		Favours experimental

**Bias in selection of the reported result**		
Is the reported effect estimate likely to be selected, on the basis of the results, from...		
7.1. ... multiple outcome *measurements* within the outcome domain?	All 13 knowledge/self-efficacy items reported in Table 2	PN
7.2 ... multiple *analyses* of the intervention-outcome relationship?	Only paired t-test reported, no evidence of multiple analyses	PN
7.3 ... different *subgroups*?	Subgroup analysis by language (Korean vs English) reported, but pre-specified as the acculturation hypothesis	PY
**Risk of bias judgement**	No registered protocol, but reporting appears complete. Subgroup analyses align with stated secondary hypothesis (acculturation).	Moderate
Optional: What is the predicted direction of bias due to selection of the reported result?		Unpredictable

**Overall bias**		
**Risk of bias judgement**	Single-arm pre-post design, self-reported outcomes measured minutes after teaching, convenience sample, no sensitivity analysis, no registered protocol, though reporting appears complete.	Serious
Optional: What is the overall predicted direction of bias for this outcome?		Favours experimental /

This work is licensed under a Creative Commons Attribution-NonCommercial-NoDerivatives 4.0 International License.

Study ID: Hoeft et al. 2016 [Ref 44]

ROBINS-I tool (Stage II): For each study

Specify a target randomized trial specific to the study

Design	Individually randomized
Participants	Low-income Spanish-speaking parents/caregivers of children aged 0–5 years; predominantly Mexican-born mothers, rural California
Experimental intervention	Contra Caries Oral Health Education Program (CCOHEP): 4 x 2-hour interactive classes led by trained promotors covering toothbrushing, caries etiology, nutrition, and dental visits
Comparator	Usual care (no CCOHEP)

Is your aim for this study…?

- ☐ to assess the effect of *assignment to* intervention
- ☐ to assess the effect of *starting and adhering to* intervention

Specify the outcome

Specify which outcome is being assessed for risk of bias (typically from among those earmarked for the Summary of Findings table). Specify whether this is a proposed benefit or harm of intervention.

Performing all 5 recommended toothbrushing behaviours (composite binary score, primary outcome)

Specify the numerical result being assessed

In case of multiple alternative analyses being presented, specify the numeric result (e.g. RR = 1.52 (95% CI 0.83 to 2.77) and/or a reference (e.g. to a table, figure or paragraph) that uniquely defines the result being assessed.

13% at baseline → 44% at post-test 1, P < 0.001 (Table 2)

Preliminary consideration of confounders

Complete a row for each important confounding domain (i) listed in the review protocol; and (ii) relevant to the setting of this particular study, or which the study authors identified as potentially important.

"Important" confounding domains are those for which, in the context of this study, adjustment is expected to lead to a clinically important change in the estimated effect of the intervention. "Validity" refers to whether the confounding variable or variables fully measure the domain, while "reliability" refers to the precision of the measurement (more measurement error means less reliability).

**(i) Confounding domains listed in the review protocol**				
Confounding domain	Measured variable(s)	Is there evidence that controlling for this variable was unnecessary?*	Is the confounding domain measured validly and reliably by this variable (or these variables)?	OPTIONAL: Is failure to adjust for this variable (alone) expected to favour the experimental intervention or the comparator?
Baseline knowledge	BRFQ pre-test	No	Yes	Favour experimental
Baseline behaviours	Composite score pre-test	No	Yes	Favour experimental
Child age	Caregiver-reported	No	Yes	No information
SES	Income, WIC receipt	No	Yes	No information
Parental education	Years schooling	No	Yes	No information
Language proficiency	Inclusion criterion (Spanish only)	No	Yes	N/A
Financial incentives	$5/session + $20–30/survey	No	Yes	Favour experimental

(ii) Additional confounding domains relevant to the setting of this particular study, or which the study authors identified as important				
Confounding domain	Measured variable(s)	Is there evidence that controlling for this variable was unnecessary?*	Is the confounding domain measured validly and reliably by this variable (or these variables)?	OPTIONAL: Is failure to adjust for this variable (alone) expected to favour the experimental intervention or the comparator?
Promotora variability	4 promotoras, ≥2 classes each	No	No	No information
Child caries status at baseline	Not measured clinically	No	No	No information
Fluoride varnish receipt	48% received in past year	No	Yes	Favour experimental
Follow-up length (4 months)	Timeline documented	No	No	Favour experimental
Social desirability	Not measured	No	Yes	Favour experimental
Rolling enrolment (13 classes)	Documented	No	Yes	No information

* In the context of a particular study, variables can be demonstrated not to be confounders and so not included in the analysis: (a) if they are not predictive of the outcome; (b) if they are not predictive of intervention; or (c) because adjustment makes no or minimal difference to the estimated effect of the primary parameter. Note that "no statistically significant association" is not the same as "not predictive".

Preliminary consideration of co-interventions

Complete a row for each important co-intervention (i) listed in the review protocol; and (ii) relevant to the setting of this particular study, or which the study authors identified as important.

"Important" co-interventions are those for which, in the context of this study, adjustment is expected to lead to a clinically important change in the estimated effect of the intervention.

**(i) Co-interventions listed in the review protocol**		
Co-intervention	Is there evidence that controlling for this co-intervention was unnecessary (e.g. because it was not administered)?	Is presence of this co-intervention likely to favour outcomes in the experimental intervention or the comparator
Routine dental care during study	No	Favour experimental
Other community health programmes	No	Favour experimental
Home visiting services	No	Favour experimental

**(ii) Additional co-interventions relevant to the setting of this particular study, or which the study authors identified as important**		
Co-intervention	Is there evidence that controlling for this co-intervention was unnecessary (e.g. because it was not administered)?	Is presence of this co-intervention likely to favour outcomes in the experimental intervention or the comparator
Fluoride varnish (48% received in past year)	No	Favour experimental
Free toothbrushes/toothpaste per session	No	Favour experimental
Financial incentives ($5/session, $20–30/survey)	No	Favour experimental

Risk of bias assessment

Responses underlined in green are potential markers for low risk of bias, and responses in red are potential markers for a risk of bias. Where questions relate only to sign posts to other questions, no formatting is used.

Signalling questions	Description	Response options
**Bias due to confounding**		
1.1 Is there potential for confounding of the effect of intervention in this study?<br>**If N/PN to 1.1:** the study can be considered to be at low risk of bias due to confounding and no further signalling questions need be considered	Single arm, no control, 4-month follow-up, multiple unadjusted confounders (incentives, fluoride varnish, free supplies, promotora variability)	Y
**If Y/PY to 1.1**: determine whether there is a need to assess time-varying confounding:		
1.2. Was the analysis based on splitting participants' follow up time according to intervention received?<br>**If N/PN**, answer questions relating to baseline confounding (1.4 to 1.6)<br>**If Y/PY**, go to question 1.3.	McNemar's test for paired pre/post only; no time-split analysis	N
1.3. Were intervention discontinuations or switches likely to be related to factors that are prognostic for the outcome?<br>**If N/PN**, answer questions relating to baseline confounding (1.4 to 1.6)<br>**If Y/PY**, answer questions relating to both baseline and time-varying confounding (1.7 and 1.8)		NA

**Questions relating to baseline confounding only**		
1.4. Did the authors use an appropriate analysis method that controlled for all the important confounding domains?	McNemar's test only; no adjustment for incentives, fluoride varnish, promotora variability, or free supplies	N / NI
1.5. **If Y/PY to 1.4**: Were confounding domains that were controlled for measured validly and reliably by the variables available in this study?		NA
1.6. Did the authors control for any post-intervention variables that could have been affected by the intervention?	No post-intervention variables included in model	NA
**Questions relating to baseline and time-varying confounding**		
1.7. Did the authors use an appropriate analysis method that controlled for all the important confounding domains and for time-varying confounding?		NA
1.8. **If Y/PY to 1.7**: Were confounding domains that were controlled for measured validly and reliably by the variables available in this study?		NA
**Risk of bias judgement**	Single-arm design with no control group, multiple unadjusted co-interventions (fluoride varnish, free supplies, financial incentives), and promotor variability across 13 classes uncontrolled.	Serious
Optional: What is the predicted direction of bias due to confounding?		Favours experimental

Bias in selection of participants into the study		
2.1. Was selection of participants into the study (or into the analysis) based on participant characteristics observed after the start of intervention?<br>**If N/PN to 2.1:** go to 2.4	Eligibility applied at baseline only (age, income, language, Hispanic identity)	N
2.2. **If Y/PY to 2.1**: Were the post-intervention variables that influenced selection likely to be associated with intervention?		NA
2.3 **If Y/PY to 2.2**: Were the post-intervention variables that influenced selection likely to be influenced by the outcome or a cause of the outcome?		NA
2.4. Do start of follow-up and start of intervention coincide for most participants?	Baseline pretest administered at first class session	Y
2.5. **If Y/PY to 2.2 and 2.3, or N/PN to 2.4**: Were adjustment techniques used that are likely to correct for the presence of selection biases?		NA
**Risk of bias judgement**	Clear eligibility criteria applied at baseline, rolling enrolment documented, and follow-up started simultaneously with intervention.	Low
Optional: What is the predicted direction of bias due to selection of participants into the study?		Unpredictable

Bias in classification of interventions		
3.1 Were intervention groups clearly defined?	CCOHEP well-described: 4 x 2-hour sessions, standardised curriculum, trained promotors	Y
3.2 Was the information used to define intervention groups recorded at the start of the intervention?	Baseline pretest and enrolment documented at first session	Y
3.3 Could classification of intervention status have been affected by knowledge of the outcome or risk of the outcome?	Single arm; all received CCOHEP, no misclassification possible	N
**Risk of bias judgement**	Intervention clearly defined, standardised across 13 classes, and consistently documented.	Low
Optional: What is the predicted direction of bias due to classification of interventions?		Unpredictable

Bias due to deviations from intended interventions		
**If your aim for this study is to assess the effect of assignment to intervention, answer questions 4.1 and 4.2**		
4.1. Were there deviations from the intended intervention beyond what would be expected in usual practice?	89% attended all 4 sessions; missed classes due to illness only; standardised curriculum across all 13 classes	PN
4.2. **If Y/PY to 4.1**: Were these deviations from intended intervention unbalanced between groups *and* likely to have affected the outcome?		NA
**If your aim for this study is to assess the effect of starting and adhering to intervention, answer questions 4.3 to 4.6**		
4.3. Were important co-interventions balanced across intervention groups?		NA
4.4. Was the intervention implemented successfully for most participants?		NA
4.5. Did study participants adhere to the assigned intervention regimen?		NA
4.6. **If N/PN to 4.3, 4.4 or 4.5**: Was an appropriate analysis used to estimate the effect of starting and adhering to the intervention?		NA
**Risk of bias judgement**	High attendance (89% completed all 4 sessions), standardised curriculum, and deviations explained by illness rather than outcome-related factors.	Low
Optional: What is the predicted direction of bias due to deviations from the intended interventions?		Unpredictable

**Bias due to missing data**		
5.1 Were outcome data available for all, or nearly all, participants?	Moderate attrition by post-test 2	**PY** for post-test 1 (90%); PN for post-test 2 (75%)
5.2 Were participants excluded due to missing data on intervention status?	Single arm, no intervention status missing	N
5.3 Were participants excluded due to missing data on other variables needed for the analysis?	26 missing at post-test 2; reasons documented (sick, unavailable)	Y
5.4 **If PN/N to 5.1, or Y/PY to 5.2 or 5.3**: Are the proportion of participants and reasons for missing data similar across interventions?	No baseline differences between completers and dropouts on age, education, behaviours	Y
5.5 **If PN/N to 5.1, or Y/PY to 5.2 or 5.3**: Is there evidence that results were robust to the presence of missing data?	Sensitivity analyses conducted: setting missing = previous value showed no significant changes; setting missing = baseline values changed only 2 minor items	PY
**Risk of bias judgement**	25% attrition at post-test 2 is notable, but sensitivity analyses were conducted and dropout was unrelated to baseline characteristics or outcomes, reducing bias concern.	Moderate
Optional: What is the predicted direction of bias due to missing data?		Favours experimental

Bias in measurement of outcomes		
6.1 Could the outcome measure have been influenced by knowledge of the intervention received?	Self-reported behaviours; parents knew intervention goals and correct answers after attending classes	Y
6.2 Were outcome assessors aware of the intervention received by study participants?	Separate bilingual researchers administered surveys, NOT the promotors who taught classes	PN
6.3 Were the methods of outcome assessment comparable across intervention groups?	Single arm	NI
6.4 Were any systematic errors in measurement of the outcome related to intervention received?	Social desirability likely despite separate assessors; authors acknowledge this explicitly	PY
**Risk of bias judgement**	Participants still knew the correct answers from the classes, making social desirability unavoidable in self-report outcomes.	Moderate
Optional: What is the predicted direction of bias due to measurement of outcomes?		Favours experimental

Bias in selection of the reported result		
Is the reported effect estimate likely to be selected, on the basis of the results, from...	All outcomes reported including non-significant ones (sweet foods, bottle use, adult assistance at post-test 1)	
7.1. ... multiple outcome *measurements* within the outcome domain?	Consistent use of McNemar's test throughout; no evidence of switching methods	PN
7.2 ... multiple *analyses* of the intervention-outcome relationship?	No subgroup analyses reported; demographic factors tested as covariates but all non-significant and fully reported	PN
7.3 ... different *subgroups*?		PN
**Risk of bias judgement**	Complete reporting of all outcomes including non-significant results, consistent analysis method, and no selective subgroup reporting. No registered protocol identified but reporting appears transparent.	Low
Optional: What is the predicted direction of bias due to selection of the reported result?		Unpredictable

**Overall bias**		
**Risk of bias judgement**	Hoeft 2016 was rated at serious overall risk of bias due to its single-arm design with no control group and multiple unadjusted co-interventions including financial incentives, free supplies, and fluoride varnish receipt. Although separate researchers administered surveys reducing measurement bias, self-reported outcomes remain susceptible to social desirability, and 25% attrition at 3-month follow-up was not fully resolved despite sensitivity analyses.	Serious
Optional: What is the overall predicted direction of bias for this outcome?		Favours experimental

This work is licensed under a Creative Commons Attribution-NonCommercial-NoDerivatives 4.0 International License.

Study ID: Lumsden et al. 2019 [Ref 35]

ROBINS-I tool (Stage II): For each study

Specify a target randomized trial specific to the study

Design	Individually randomized
Participants	Low-income Hispanic parent/child dyads (children 2–6 yrs), NYC dental clinic
Experimental intervention	Single session iPad-based MySmileBuddy ECC education + goal-setting (~15–20 mins)
Comparator	No comparator

Is your aim for this study…?

☐ to assess the effect of *assignment to* intervention

☐ to assess the effect of *starting and adhering to* intervention

Specify the outcome

Specify which outcome is being assessed for risk of bias (typically from among those earmarked for the Summary of Findings table). Specify whether this is a proposed benefit or harm of intervention.

Self-reported behaviour change re: diet and oral hygiene (proposed benefit); assessed at 1-month telephone follow-up

Specify the numerical result being assessed

In case of multiple alternative analyses being presented, specify the numeric result (e.g. RR = 1.52 (95% CI 0.83 to 2.77) and/or a reference (e.g. to a table, figure or paragraph) that uniquely defines the result being assessed.

79.7% (n=63/79) reported taking some action to change behaviour post-intervention (Table 1)

Preliminary consideration of confounders

Complete a row for each important confounding domain (i) listed in the review protocol; and (ii) relevant to the setting of this particular study, or which the study authors identified as potentially important.

"Important" confounding domains are those for which, in the context of this study, adjustment is expected to lead to a clinically important change in the estimated effect of the intervention. "Validity" refers to whether the confounding variable or variables fully measure the domain, while "reliability" refers to the precision of the measurement (more measurement error means less reliability).

**(i) Confounding domains listed in the review protocol**				
Confounding domain	Measured variable(s)	Is there evidence that controlling for this variable was unnecessary?*	Is the confounding domain measured validly and reliably by this variable (or these variables)?	OPTIONAL: Is failure to adjust for this variable (alone) expected to favour the experimental intervention or the comparator?
Baseline OH knowledge/behaviour	Not measured	No	No information	No information
SES	SNAP/Medicaid status	No	Yes	No information
Education level	Self-reported	No	Yes	No information
Dental clinic attendance (pre-existing OH interest)	Not measured	No	No information	Favour experimental
History/maturation effects	No control group	No	No	Favour experimental

**(ii) Additional confounding domains relevant to the setting of this particular study, or which the study authors identified as important**				
Confounding domain	Measured variable(s)	Is there evidence that controlling for this variable was unnecessary?*	Is the confounding domain measured validly and reliably by this variable (or these variables)?	OPTIONAL: Is failure to adjust for this variable (alone) expected to favour the experimental intervention or the comparator?
Routine dental provider counselling during same visit	Not measured	No	No	Favour experimental

* In the context of a particular study, variables can be demonstrated not to be confounders and so not included in the analysis: (a) if they are not predictive of the outcome; (b) if they are not predictive of intervention; or (c) because adjustment makes no or minimal difference to the estimated effect of the primary parameter. Note that "no statistically significant association" is not the same as "not predictive".

Preliminary consideration of co-interventions

Complete a row for each important co-intervention (i) listed in the review protocol; and (ii) relevant to the setting of this particular study, or which the study authors identified as important.

"Important" co-interventions are those for which, in the context of this study, adjustment is expected to lead to a clinically important change in the estimated effect of the intervention.

**(i) Co-interventions listed in the review protocol**		
Co-intervention	Is there evidence that controlling for this co-intervention was unnecessary (e.g. because it was not administered)?	Is presence of this co-intervention likely to favour outcomes in the experimental intervention or the comparator
Dental attendance during study	No	No information
General health education outside study	No	No information
OH materials provided	No	No information

**(ii) Additional co-interventions relevant to the setting of this particular study, or which the study authors identified as important**		
Co-intervention	Is there evidence that controlling for this co-intervention was unnecessary (e.g. because it was not administered)?	Is presence of this co-intervention likely to favour outcomes in the experimental intervention or the comparator
Routine dental provider OH counselling during same visit	No / acknowledged in limitations	Favour experimental
$10 transit card (participation incentive)	No	Favour experimental

Risk of bias assessment

Responses underlined in green are potential markers for low risk of bias, and responses in red are potential markers for a risk of bias. Where questions relate only to sign posts to other questions, no formatting is used.

Signalling questions	Description	Response options
**Bias due to confounding**		
1.1 Is there potential for confounding of the effect of intervention in this study?<br>**If N/PN to 1.1:** the study can be considered to be at low risk of bias due to confounding and no further signalling questions need be considered	no control group; routine dental counselling confounds results; pre-existing OH interest in clinic attendees	Y
**If Y/PY to 1.1**: determine whether there is a need to assess time-varying confounding:		
1.2. Was the analysis based on splitting participants' follow up time according to intervention received?<br>**If N/PN**, answer questions relating to baseline confounding (1.4 to 1.6)<br>**If Y/PY**, go to question 1.3.	single pre-post, one follow-up point only	NA
1.3. Were intervention discontinuations or switches likely to be related to factors that are prognostic for the outcome?<br>**If N/PN**, answer questions relating to baseline confounding (1.4 to 1.6)<br>**If Y/PY**, answer questions relating to both baseline and time-varying confounding (1.7 and 1.8)		NA

**Questions relating to baseline confounding only**		
1.4. Did the authors use an appropriate analysis method that controlled for all the important confounding domains?	descriptive statistics only, no adjustment attempted	N
1.5. **If Y/PY to 1.4**: Were confounding domains that were controlled for measured validly and reliably by the variables available in this study?	no control group, no adjustment	N
1.6. Did the authors control for any post-intervention variables that could have been affected by the intervention?	routine dental counselling during same visit not controlled	N
**Questions relating to baseline and time-varying confounding**		
1.7. Did the authors use an appropriate analysis method that controlled for all the important confounding domains and for time-varying confounding?		NA
1.8. **If Y/PY to 1.7**: Were confounding domains that were controlled for measured validly and reliably by the variables available in this study?		NA
**Risk of bias judgement**		Serious
Optional: What is the predicted direction of bias due to confounding?		Favours experimental

Bias in selection of participants into the study		
2.1. Was selection of participants into the study (or into the analysis) based on participant characteristics observed after the start of intervention?<br>**If N/PN to 2.1:** go to 2.4	recruited before intervention based on clinic attendance	N
2.2. **If Y/PY to 2.1**: Were the post-intervention variables that influenced selection likely to be associated with intervention?		NA / Y / PY / PN / N / NI
2.3 **If Y/PY to 2.2**: Were the post-intervention variables that influenced selection likely to be influenced by the outcome or a cause of the outcome?		NA / Y / PY / PN / N / NI
2.4. Do start of follow-up and start of intervention coincide for most participants?	intervention and baseline assessment simultaneous at clinic visit	Y
2.5. **If Y/PY to 2.2 and 2.3, or N/PN to 2.4**: Were adjustment techniques used that are likely to correct for the presence of selection biases?		NA
**Risk of bias judgement**	Dental clinic sample self-selected, already health-seeking, likely higher OH motivation than general CALD population. No control group.	Serious
Optional: What is the predicted direction of bias due to selection of participants into the study?		Favours experimental

Bias in classification of interventions		
3.1 Were intervention groups clearly defined?	single MySmileBuddy session, protocol clearly described	Y
3.2 Was the information used to define intervention groups recorded at the start of the intervention?	administered at clinic visit, pre-specified protocol	Y
3.3 Could classification of intervention status have been affected by knowledge of the outcome or risk of the outcome?	single group, no misclassification possible	N
**Risk of bias judgement**		Low
Optional: What is the predicted direction of bias due to classification of interventions?		N/A

Bias due to deviations from intended interventions		
**If your aim for this study is to assess the effect of assignment to intervention, answer questions 4.1 and 4.2**		
4.1. Were there deviations from the intended intervention beyond what would be expected in usual practice?	Routine dental provider counselling occurred alongside intervention; inseparable co-intervention	PN
4.2. **If Y/PY to 4.1**: Were these deviations from intended intervention unbalanced between groups *and* likely to have affected the outcome?	No comparator so imbalance cannot be assessed	PN
**If your aim for this study is to assess the effect of starting and adhering to intervention, answer questions 4.3 to 4.6**		
4.3. Were important co-interventions balanced across intervention groups?		Y / PY / PN / N / NI
4.4. Was the intervention implemented successfully for most participants?		Y / PY / PN / N / NI
4.5. Did study participants adhere to the assigned intervention regimen?		Y / PY / PN / N / NI
4.6. **If N/PN to 4.3, 4.4 or 4.5**: Was an appropriate analysis used to estimate the effect of starting and adhering to the intervention?		NA
**Risk of bias judgement**		Moderate
Optional: What is the predicted direction of bias due to deviations from the intended interventions?		Favours experimental

Bias due to missing data		
5.1 Were outcome data available for all, or nearly all, participants?	73.1% reached at follow-up; 26.9% attrition	PN
5.2 Were participants excluded due to missing data on intervention status?	29 lost to follow-up, reasons documented but unresolved	PN
5.3 Were participants excluded due to missing data on other variables needed for the analysis?	same 29 excluded	PN
5.4 **If PN/N to 5.1, or Y/PY to 5.2 or 5.3**: Are the proportion of participants and reasons for missing data similar across interventions?	demographic comparison showed no significant differences between lost and retained	PY
5.5 **If PN/N to 5.1, or Y/PY to 5.2 or 5.3**: Is there evidence that results were robust to the presence of missing data?	no sensitivity analysis conducted	PN
**Risk of bias judgement**		Moderate
Optional: What is the predicted direction of bias due to missing data?		Favours experimental

Bias in measurement of outcomes		
6.1 Could the outcome measure have been influenced by knowledge of the intervention received?	self-reported behaviour change, social desirability bias likely	Y
6.2 Were outcome assessors aware of the intervention received by study participants?	same investigators administered intervention and follow-up survey	Y
6.3 Were the methods of outcome assessment comparable across intervention groups?	no comparator	N
6.4 Were any systematic errors in measurement of the outcome related to intervention received?	structured telephone script used but self-report only	PN
**Risk of bias judgement**		Serious
Optional: What is the predicted direction of bias due to measurement of outcomes?		Favours experimental

Bias in selection of the reported result		
Is the reported effect estimate likely to be selected, on the basis of the results, from... 7.1. ... multiple outcome *measurements* within the outcome domain?	single follow-up timepoint only	N
7.2 ... multiple *analyses* of the intervention-outcome relationship?	descriptive statistics only, consistent throughout	Y / PY / PN / N / NI
7.3 ... different *subgroups*?	no subgroup analyses	Y / PY / PN / N / NI
**Risk of bias judgement**		Low
Optional: What is the predicted direction of bias due to selection of the reported result?		N/A

Overall bias		
**Risk of bias judgement**	No control group, high attrition at follow-up (26.9%), and self-reported outcomes assessed by same investigators who delivered the intervention. Results cannot be attributed to the intervention alone.	Serious
Optional: What is the overall predicted direction of bias for this outcome?		Favours experimental

This work is licensed under a Creative Commons Attribution-NonCommercial-NoDerivatives 4.0 International License.

Study ID: Yuan et al. 2019 [Ref 42]

ROBINS-I tool (Stage II): For each study

Specify a target randomized trial specific to the study

Design	Individually randomized
Participants	Chinese undocumented migrant mothers with infants aged <8 weeks, residing in Belfast, Northern Ireland
Experimental intervention	Community-based culturally tailored oral health education via home visits and telephone calls over 12 months, delivered by a Chinese community health worker
Comparator	No oral health education intervention (control group, matched on demographics)

Is your aim for this study…?

☐ to assess the effect of *assignment to* intervention

☐ to assess the effect of *starting and adhering to* intervention

Specify the outcome

Specify which outcome is being assessed for risk of bias (typically from among those earmarked for the Summary of Findings table). Specify whether this is a proposed benefit or harm of intervention.

Maternal oral health knowledge, attitudes, and self-reported behaviours regarding baby toothbrushing and sugar snacking (proposed benefit of intervention); assessed at 8 weeks, 6 months, and 12 months

Specify the numerical result being assessed

In case of multiple alternative analyses being presented, specify the numeric result (e.g. RR = 1.52 (95% CI 0.83 to 2.77) and/or a reference (e.g. to a table, figure or paragraph) that uniquely defines the result being assessed.

Knowledge at 12 months: $\chi^2 = 14.12$, $p = 0.004$ (Table in Results, Section 3.1); Attitudinal change scores: Table 4

Preliminary consideration of confounders

Complete a row for each important confounding domain (i) listed in the review protocol; and (ii) relevant to the setting of this particular study, or which the study authors identified as potentially important.

"Important" confounding domains are those for which, in the context of this study, adjustment is expected to lead to a clinically important change in the estimated effect of the intervention. "Validity" refers to whether the confounding variable or variables fully measure the domain, while "reliability" refers to the precision of the measurement (more measurement error means less reliability).

**(i) Confounding domains listed in the review protocol**				
Confounding domain	Measured variable(s)	Is there evidence that controlling for this variable was unnecessary?*	Is the confounding domain measured validly and reliably by this variable (or these variables)?	OPTIONAL: Is failure to adjust for this variable (alone) expected to favour the experimental intervention or the comparator?
Baseline OH knowledge	Questionnaire item at 8 weeks	No	Yes	No information
SES / deprivation	Noble Index (postcode)	No	Yes	No information
Maternal education	Years of education (≤12 or >12)	No	Yes	No information
No. of children	Self-reported	No	Yes	Favour comparator
Temporal recruitment gap	Control recruited 6 months earlier	No	No	Favour experimental
Language proficiency	Not measured	No	No information	No information

(ii) Additional confounding domains relevant to the setting of this particular study, or which the study authors identified as important				
Confounding domain	Measured variable(s)	Is there evidence that controlling for this variable was unnecessary?*	Is the confounding domain measured validly and reliably by this variable (or these variables)?	OPTIONAL: Is failure to adjust for this variable (alone) expected to favour the experimental intervention or the comparator?
Researcher = interventionist + data collector	Not measured	No	No	Favour experimental
Control group contamination (SY gave advice to controls)	Not quantified	No	No	Favour comparator

* In the context of a particular study, variables can be demonstrated not to be confounders and so not included in the analysis: (a) if they are not predictive of the outcome; (b) if they are not predictive of intervention; or (c) because adjustment makes no or minimal difference to the estimated effect of the primary parameter. Note that "no statistically significant association" is not the same as "not predictive".

Preliminary consideration of co-interventions

Complete a row for each important co-intervention (i) listed in the review protocol; and (ii) relevant to the setting of this particular study, or which the study authors identified as important.

"Important" co-interventions are those for which, in the context of this study, adjustment is expected to lead to a clinically important change in the estimated effect of the intervention.

**(i) Co-interventions listed in the review protocol**		
Co-intervention	Is there evidence that controlling for this co-intervention was unnecessary (e.g. because it was not administered)?	Is presence of this co-intervention likely to favour outcomes in the experimental intervention or the comparator
Dental service attendance during study	No	Favour experimental
Receipt of OH materials (toothbrush/toothpaste)	No	Favour experimental
General health/home visiting services outside study	No	No information

**(ii) Additional co-interventions relevant to the setting of this particular study, or which the study authors identified as important**		
Co-intervention	Is there evidence that controlling for this co-intervention was unnecessary (e.g. because it was not administered)?	Is presence of this co-intervention likely to favour outcomes in the experimental intervention or the comparator
Social support provided alongside OH education (SY)	No bundled with intervention, inseparable	Favour experimental

Risk of bias assessment

Responses underlined in green are potential markers for low risk of bias, and responses in red are potential markers for a risk of bias. Where questions relate only to sign posts to other questions, no formatting is used.

Signalling questions	Description	Response options
**Bias due to confounding**		
1.1 Is there potential for confounding of the effect of intervention in this study?<br>**If N/PN to 1.1:** the study can be considered to be at low risk of bias due to confounding and no further signalling questions need be considered	Non-random allocation, temporal recruitment gap, researcher as interventionist, contamination.	Y
**If Y/PY to 1.1**: determine whether there is a need to assess time-varying confounding:		
1.2. Was the analysis based on splitting participants' follow up time according to intervention received?<br>**If N/PN**, answer questions relating to baseline confounding (1.4 to 1.6)<br>**If Y/PY**, go to question 1.3.	Groups compared at fixed time points (8 weeks, 6 months, 12 months), not split by time receiving intervention.	N
1.3. Were intervention discontinuations or switches likely to be related to factors that are prognostic for the outcome?<br>**If N/PN**, answer questions relating to baseline confounding (1.4 to 1.6)<br>**If Y/PY**, answer questions relating to both baseline and time-varying confounding (1.7 and 1.8)		NA

**Questions relating to baseline confounding only**		
1.4. Did the authors use an appropriate analysis method that controlled for all the important confounding domains?	Only chi-square and t-tests used. No regression or covariate adjustment.	PN
1.5. **If Y/PY to 1.4**: Were confounding domains that were controlled for measured validly and reliably by the variables available in this study?	Matching was attempted but temporal gap and researcher role were uncontrolled.	PN
1.6. Did the authors control for any post-intervention variables that could have been affected by the intervention?	No adjustment for contamination or co-interventions (social support bundled with OH education	N
**Questions relating to baseline and time-varying confounding**		
1.7. Did the authors use an appropriate analysis method that controlled for all the important confounding domains and for time-varying confounding?		NA
1.8. **If Y/PY to 1.7**: Were confounding domains that were controlled for measured validly and reliably by the variables available in this study?		NA
**Risk of bias judgement**	Uncontrolled confounders (researcher role, temporal gap, bundled social support) all lean toward overestimating the intervention effect.	Moderate
Optional: What is the predicted direction of bias due to confounding?		Favours experimental / Favours comparator / Unpredictable

Bias in selection of participants into the study		
2.1. Was selection of participants into the study (or into the analysis) based on participant characteristics observed after the start of intervention?<br>**If N/PN to 2.1:** go to 2.4	Selection was based on pre-intervention matching (age, education, deprivation). No post-intervention selection criteria used.	PN
2.2. **If Y/PY to 2.1**: Were the post-intervention variables that influenced selection likely to be associated with intervention?		NA
2.3 **If Y/PY to 2.2**: Were the post-intervention variables that influenced selection likely to be influenced by the outcome or a cause of the outcome?		NA
2.4. Do start of follow-up and start of intervention coincide for most participants?	Both groups assessed from 8 weeks infant age. Intervention started at same point for intervention group.	PY
2.5. **If Y/PY to 2.2 and 2.3, or N/PN to 2.4**: Were adjustment techniques used that are likely to correct for the presence of selection biases?		NA
**Risk of bias judgement**	Convenience snowball sampling, non-random allocation, and the 6-month temporal recruitment gap all introduce selection bias favouring the intervention group (newer recruits, potentially more motivated).	Moderate
Optional: What is the predicted direction of bias due to selection of participants into the study?		Favours experimental / Favours comparator / Towards null /Away from null / Unpredictable

Bias in classification of interventions		
3.1 Were intervention groups clearly defined?		Y
3.2 Was the information used to define intervention groups recorded at the start of the intervention?		Y
3.3 Could classification of intervention status have been affected by knowledge of the outcome or risk of the outcome?		N
**Risk of bias judgement**	Intervention and comparator are clearly and consistently defined, recorded prospectively, and not subject to misclassification.	Low
Optional: What is the predicted direction of bias due to classification of interventions?		Favours experimental / Favours comparator / Towards null /Away from null / Unpredictable

Bias due to deviations from intended interventions		
**If your aim for this study is to assess the effect of assignment to intervention, answer questions 4.1 and 4.2**		
4.1. Were there deviations from the intended intervention beyond what would be expected in usual practice?	Two issues: (1) control mothers received some dental advice from SY for ethical reasons (contamination); (2) social support was provided alongside OH education, beyond what usual practice would include.	Y
4.2. **If Y/PY to 4.1**: Were these deviations from intended intervention unbalanced between groups *and* likely to have affected the outcome?	Contamination affected controls (diluting effect); extra social support benefited intervention group only. Both unbalanced and outcome-relevant.	Y
**If your aim for this study is to assess the effect of starting and adhering to intervention, answer questions 4.3 to 4.6**		
4.3. Were important co-interventions balanced across intervention groups?		NA
4.4. Was the intervention implemented successfully for most participants?		NA
4.5. Did study participants adhere to the assigned intervention regimen?		NA
4.6. **If N/PN to 4.3, 4.4 or 4.5**: Was an appropriate analysis used to estimate the effect of starting and adhering to the intervention?		NA
**Risk of bias judgement**		Moderate
Optional: What is the predicted direction of bias due to deviations from the intended interventions?		Favours experimental

Bias due to missing data		
5.1 Were outcome data available for all, or nearly all, participants?	2/36 lost to follow-up (one per group, both moved from NI).	Y
5.2 Were participants excluded due to missing data on intervention status?		PN
5.3 Were participants excluded due to missing data on other variables needed for the analysis?		PN
5.4 **If PN/N to 5.1, or Y/PY to 5.2 or 5.3**: Are the proportion of participants and reasons for missing data similar across interventions?		NA
5.5 **If PN/N to 5.1, or Y/PY to 5.2 or 5.3**: Is there evidence that results were robust to the presence of missing data?		NA
**Risk of bias judgement**	Attrition balanced, minimal and equally distributed.	Low
Optional: What is the predicted direction of bias due to missing data?		Unpredictable

Bias in measurement of outcomes		
6.1 Could the outcome measure have been influenced by knowledge of the intervention received?	Primary outcomes are self-reported. Social desirability bias likely — mothers knew which group they were in.	Y
6.2 Were outcome assessors aware of the intervention received by study participants?	SY delivered the intervention AND collected questionnaire data. Only dental examiner (AS) was blinded.	Y
6.3 Were the methods of outcome assessment comparable across intervention groups?	Same questionnaire used for both groups.	Y
6.4 Were any systematic errors in measurement of the outcome related to intervention received?	Self-report by unblinded participants + SY as both interventionist and assessor = serious detection bias risk.	PY
**Risk of bias judgement**		Serious
Optional: What is the predicted direction of bias due to measurement of outcomes?		Favours experimental

Bias in selection of the reported result		
Is the reported effect estimate likely to be selected, on the basis of the results, from...		
7.1. ... multiple outcome *measurements* within the outcome domain?	Outcomes measured at 3 time points (8 weeks, 6 months, 12 months) all reported, not selectively picked.	PN
7.2 ... multiple *analyses* of the intervention-outcome relationship?	Chi-square and t-tests used consistently.	PN
7.3 ... different *subgroups*?		N
**Risk of bias judgement**		Low
Optional: What is the predicted direction of bias due to selection of the reported result?		Unpredictable

Overall bias		
**Risk of bias judgement**		Serious
Optional: What is the overall predicted direction of bias for this outcome?		Favours experimental

This work is licensed under a Creative Commons Attribution-NonCommercial-NoDerivatives 4.0 International License.

Study ID: Ponce-Gonzalez et al. 2019 [Ref 43]

ROBINS-I tool (Stage II): For each study

Specify a target randomized trial specific to the study

Design	Pre-post uncontrolled
Participants	Latino migrant farmworkers, Washington State, 2017
Experimental intervention	2-hour interactive lay-led OH education (12 workshops)
Comparator	No comparator

Is your aim for this study…?

- ☐ to assess the effect of *assignment to* intervention
- ☐ to assess the effect of *starting and adhering to* intervention

Specify the outcome

Specify which outcome is being assessed for risk of bias (typically from among those earmarked for the Summary of Findings table). Specify whether this is a proposed benefit or harm of intervention.

OH, knowledge scores (proposed benefit); self-reported via pre-post survey

Specify the numerical result being assessed

In case of multiple alternative analyses being presented, specify the numeric result (e.g. RR = 1.52 (95% CI 0.83 to 2.77) and/or a reference (e.g. to a table, figure or paragraph) that uniquely defines the result being assessed.

All 8 knowledge questions improved significantly (p<0.01); largest gain: oral health problem factors +27.2% (Table 3)

Preliminary consideration of confounders

Complete a row for each important confounding domain (i) listed in the review protocol; and (ii) relevant to the setting of this particular study, or which the study authors identified as potentially important.

"Important" confounding domains are those for which, in the context of this study, adjustment is expected to lead to a clinically important change in the estimated effect of the intervention. "Validity" refers to whether the confounding variable or variables fully measure the domain, while "reliability" refers to the precision of the measurement (more measurement error means less reliability).

**(i) Confounding domains listed in the review protocol**				
Confounding domain	Measured variable(s)	Is there evidence that controlling for this variable was unnecessary?*	Is the confounding domain measured validly and reliably by this variable (or these variables)?	OPTIONAL: Is failure to adjust for this variable (alone) expected to favour the experimental intervention or the comparator?
Baseline OH knowledge	Pre-survey	No	Yes	No information
SES	Not measured	No	No information	No information
Education level	Self-reported	No	Yes	No information
Language proficiency	Not measured	No	No information	No information
History/maturation effects	No control group	No	Yes	Favour experimental

**(ii) Additional confounding domains relevant to the setting of this particular study, or which the study authors identified as important**				
Confounding domain	Measured variable(s)	Is there evidence that controlling for this variable was unnecessary?*	Is the confounding domain measured validly and reliably by this variable (or these variables)?	OPTIONAL: Is failure to adjust for this variable (alone) expected to favour the experimental intervention or the comparator?
Lay leader variability in survey admin	Not measured	No	No	Favour experimental

* In the context of a particular study, variables can be demonstrated not to be confounders and so not included in the analysis: (a) if they are not predictive of the outcome; (b) if they are not predictive of intervention; or (c) because adjustment makes no or minimal difference to the estimated effect of the primary parameter. Note that "no statistically significant association" is not the same as "not predictive".

Preliminary consideration of co-interventions

Complete a row for each important co-intervention (i) listed in the review protocol; and (ii) relevant to the setting of this particular study, or which the study authors identified as important.

"Important" co-interventions are those for which, in the context of this study, adjustment is expected to lead to a clinically important change in the estimated effect of the intervention.

**(i) Co-interventions listed in the review protocol**		
Co-intervention	Is there evidence that controlling for this co-intervention was unnecessary (e.g. because it was not administered)?	Is presence of this co-intervention likely to favour outcomes in the experimental intervention or the comparator
Dental attendance during study	No	No information
OH materials provided	No	No information
General health education outside study	No	No information

**(ii) Additional co-interventions relevant to the setting of this particular study, or which the study authors identified as important**		
Co-intervention	Is there evidence that controlling for this co-intervention was unnecessary (e.g. because it was not administered)?	Is presence of this co-intervention likely to favour outcomes in the experimental intervention or the comparator
$25 participant stipend (attendance incentive)	No	Favour experimental

Risk of bias assessment

Responses underlined in green are potential markers for low risk of bias, and responses in red are potential markers for a risk of bias. Where questions relate only to sign posts to other questions, no formatting is used.

Signalling questions	Description	Response options
**Bias due to confounding**		
1.1 Is there potential for confounding of the effect of intervention in this study?<br>**If N/PN to 1.1:** the study can be considered to be at low risk of bias due to confounding and no further signalling questions need be considered	no control group, history/maturation effects uncontrolled	Y
**If Y/PY to 1.1**: determine whether there is a need to assess time-varying confounding:		
1.2. Was the analysis based on splitting participants' follow up time according to intervention received?<br>**If N/PN**, answer questions relating to baseline confounding (1.4 to 1.6)<br>**If Y/PY**, go to question 1.3.	single pre-post measurement only	N
1.3. Were intervention discontinuations or switches likely to be related to factors that are prognostic for the outcome?<br>**If N/PN**, answer questions relating to baseline confounding (1.4 to 1.6)<br>**If Y/PY**, answer questions relating to both baseline and time-varying confounding (1.7 and 1.8)		NA

**Questions relating to baseline confounding only**		
1.4. Did the authors use an appropriate analysis method that controlled for all the important confounding domains?	paired t-tests only, no adjustment for any confounders	N
1.5. **If Y/PY to 1.4**: Were confounding domains that were controlled for measured validly and reliably by the variables available in this study?	no control group, no adjustment attempted	N
1.6. Did the authors control for any post-intervention variables that could have been affected by the intervention?	not addressed	N
**Questions relating to baseline and time-varying confounding**		
1.7. Did the authors use an appropriate analysis method that controlled for all the important confounding domains and for time-varying confounding?		NA
1.8. **If Y/PY to 1.7**: Were confounding domains that were controlled for measured validly and reliably by the variables available in this study?		NA
**Risk of bias judgement**		Serious
Optional: What is the predicted direction of bias due to confounding?		Favours experimental

Bias in selection of participants into the study		
2.1. Was selection of participants into the study (or into the analysis) based on participant characteristics observed after the start of intervention?		N
**If N/PN to 2.1:** go to 2.4		
2.2. **If Y/PY to 2.1**: Were the post-intervention variables that influenced selection likely to be associated with intervention?		NA / Y / PY / PN / N / NI
2.3 **If Y/PY to 2.2**: Were the post-intervention variables that influenced selection likely to be influenced by the outcome or a cause of the outcome?		NA / Y / PY / PN / N / NI
2.4. Do start of follow-up and start of intervention coincide for most participants?		Y
2.5. **If Y/PY to 2.2 and 2.3, or N/PN to 2.4**: Were adjustment techniques used that are likely to correct for the presence of selection biases?		NA
**Risk of bias judgement**	Convenience sample of self-selected workshop attendees — highly motivated, not representative. No control group to compare against.	Serious
Optional: What is the predicted direction of bias due to selection of participants into the study?		Favours experimental

Bias in classification of interventions		
3.1 Were intervention groups clearly defined?	Single intervention group, 2-hour workshop clearly described	Y
3.2 Was the information used to define intervention groups recorded at the start of the intervention?	Pre-survey collected before session began	Y
3.3 Could classification of intervention status have been affected by knowledge of the outcome or risk of the outcome?	Group status fixed, no comparator to misclassify	N
**Risk of bias judgement**		Low
Optional: What is the predicted direction of bias due to classification of interventions?		Favours experimental / Favours comparator / Towards null /Away from null / Unpredictable

Bias due to deviations from intended interventions		
**If your aim for this study is to assess the effect of assignment to intervention, answer questions 4.1 and 4.2**		
4.1. Were there deviations from the intended intervention beyond what would be expected in usual practice?	lay leaders acknowledged to have inconsistencies in survey administration; $25 stipend not usual practice	PN
4.2. **If Y/PY to 4.1**: Were these deviations from intended intervention unbalanced between groups *and* likely to have affected the outcome?	no comparator group so imbalance cannot be assessed	PN
**If your aim for this study is to assess the effect of starting and adhering to intervention, answer questions 4.3 to 4.6**		
4.3. Were important co-interventions balanced across intervention groups?		**NA** (aim = assignment, not adherence)
4.4. Was the intervention implemented successfully for most participants?		**NA** (aim = assignment, not adherence)
4.5. Did study participants adhere to the assigned intervention regimen?		**NA** (aim = assignment, not adherence)
4.6. **If N/PN to 4.3, 4.4 or 4.5**: Was an appropriate analysis used to estimate the effect of starting and adhering to the intervention?		NA / Y / PY / PN / N / NI
**Risk of bias judgement**		Moderate
Optional: What is the predicted direction of bias due to deviations from the intended interventions?		Favours experimental

Bias due to missing data		
5.1 Were outcome data available for all, or nearly all, participants?	278/311 (89%) completed both surveys	Y
5.2 Were participants excluded due to missing data on intervention status?	33 unpaired surveys excluded, reason not fully explained	PN
5.3 Were participants excluded due to missing data on other variables needed for the analysis?		PN
5.4 **If PN/N to 5.1, or Y/PY to 5.2 or 5.3**: Are the proportion of participants and reasons for missing data similar across interventions?		NA
5.5 **If PN/N to 5.1, or Y/PY to 5.2 or 5.3**: Is there evidence that results were robust to the presence of missing data?		NA
**Risk of bias judgement**		Low
Optional: What is the predicted direction of bias due to missing data?		Favours experimental / Favours comparator / Towards null /Away from null / Unpredictable

Bias in measurement of outcomes		
6.1 Could the outcome measure have been influenced by knowledge of the intervention received?	Self-report, participants knew they received training	Y
6.2 Were outcome assessors aware of the intervention received by study participants?	Lay leaders administered both pre and post surveys	Y
6.3 Were the methods of outcome assessment comparable across intervention groups?	No comparator group	N
6.4 Were any systematic errors in measurement of the outcome related to intervention received?	Inconsistencies in survey administration acknowledged by authors	PN
**Risk of bias judgement**		Moderate
Optional: What is the predicted direction of bias due to measurement of outcomes?		Favours experimental

Bias in selection of the reported result		
Is the reported effect estimate likely to be selected, on the basis of the results, from...		
7.1. ... multiple outcome *measurements* within the outcome domain?	Single post-survey timepoint only	N
7.2 ... multiple *analyses* of the intervention-outcome relationship?	Paired t-tests only, consistent throughout	N
7.3 ... different *subgroups*?	No subgroup analyses	N
**Risk of bias judgement**		Low
Optional: What is the predicted direction of bias due to selection of the reported result?		Favours experimental / Favours comparator / Towards null /Away from null / Unpredictable

Overall bias		
**Risk of bias judgement**	No control group precludes causal inference; self-selected convenience sample and self-reported outcomes measured immediately post-session inflate observed knowledge gains. Overall risk of bias is serious, favouring the experimental group.	Serious
Optional: What is the overall predicted direction of bias for this outcome?		Favours experimental

This work is licensed under a Creative Commons Attribution-NonCommercial-NoDerivatives 4.0 International License.

Study ID: Lumsden et al. 2024 [Ref 41]

ROBINS-I tool (Stage II): For each study

Specify a target randomized trial specific to the study

Design	Individually randomized
Participants	New York City children aged 24 to 72 months with clinically diagnosed caries; predominantly Hispanic, largely immigrant, Medicaid-insured families
Experimental intervention	1-year MySmileBuddy (MSB) Program: community health worker–delivered behavioural intervention using the MSB app, including 2+ in-person visits, telephone and text follow-up, and quarterly provision of oral hygiene supplies
Comparator	Usual care (no MSB intervention)

Is your aim for this study…?

☐ to assess the effect of *assignment to* intervention

☐ to assess the effect of *starting and adhering to* intervention

Specify the outcome

Specify which outcome is being assessed for risk of bias (typically from among those earmarked for the Summary of Findings table). Specify whether this is a proposed benefit or harm of intervention.

Therapeutic toothbrushing (parent-reported composite of adult-assisted brushing with fluoridated toothpaste twice daily). Proposed **benefit** of the intervention.

Specify the numerical result being assessed

In case of multiple alternative analyses being presented, specify the numeric result (e.g. RR = 1.52 (95% CI 0.83 to 2.77) and/or a reference (e.g. to a table, figure or paragraph) that uniquely defines the result being assessed.

OR = 1.79, 95% CI = 1.46 to 2.20, P < 0.001 (Table 3, post vs preintervention, n = 864)

Preliminary consideration of confounders

Complete a row for each important confounding domain (i) listed in the review protocol; and (ii) relevant to the setting of this particular study, or which the study authors identified as potentially important.

"Important" confounding domains are those for which, in the context of this study, adjustment is expected to lead to a clinically important change in the estimated effect of the intervention. "Validity" refers to whether the confounding variable or variables fully measure the domain, while "reliability" refers to the precision of the measurement (more measurement error means less reliability).

**(i) Confounding domains listed in the review protocol**				
Confounding domain	Measured variable(s)	Is there evidence that controlling for this variable was unnecessary?*	Is the confounding domain measured validly and reliably by this variable (or these variables)?	OPTIONAL: Is failure to adjust for this variable (alone) expected to favour the experimental intervention or the comparator?
Child age (maturation over 1 year)	Age at enrolment (range 0.67–6.67 y)	No	Yes	Favour experimental
Baseline behaviours	Pre-intervention survey responses	No	No information (parent self-report)	Favour experimental
Co-interventions (external dental/educational messaging)	Not measured	No	No (not controlled, acknowledged by authors)	Favour experimental
Socioeconomic status	WIC/SNAP receipt, education, employment	No	Yes	No information
Ethnicity / immigration status	Hispanic, country of birth	No	Yes	No information
Receipt of free oral hygiene supplies	Documented (quarterly distribution)	No	Yes	Favour experimental

**(ii) Additional confounding domains relevant to the setting of this particular study, or which the study authors identified as important**				
Confounding domain	Measured variable(s)	Is there evidence that controlling for this variable was unnecessary?*	Is the confounding domain measured validly and reliably by this variable (or these variables)?	OPTIONAL: Is failure to adjust for this variable (alone) expected to favour the experimental intervention or the comparator?
Free oral hygiene supplies	Not measured	No	Yes	Experimental
External dental messaging	Not measured	No	No	Experimental
Social desirability (CHW rapport)	Not measured	No	No	Experimental
Parental engagement with referrals	Not measured	No	No	Experimental

* In the context of a particular study, variables can be demonstrated not to be confounders and so not included in the analysis: (a) if they are not predictive of the outcome; (b) if they are not predictive of intervention; or (c) because adjustment makes no or minimal difference to the estimated effect of the primary parameter. Note that "no statistically significant association" is not the same as "not predictive".

Preliminary consideration of co-interventions

Complete a row for each important co-intervention (i) listed in the review protocol; and (ii) relevant to the setting of this particular study, or which the study authors identified as important.

"Important" co-interventions are those for which, in the context of this study, adjustment is expected to lead to a clinically important change in the estimated effect of the intervention.

**(i) Co-interventions listed in the review protocol**		
Co-intervention	Is there evidence that controlling for this co-intervention was unnecessary (e.g. because it was not administered)?	Is presence of this co-intervention likely to favour outcomes in the experimental intervention or the comparator
Routine dental care (standard of care)	No	Favour experimental
Other oral health education programmes	No	Favour experimental

**(ii) Additional co-interventions relevant to the setting of this particular study, or which the study authors identified as important**		
Co-intervention	Is there evidence that controlling for this co-intervention was unnecessary (e.g. because it was not administered)?	Is presence of this co-intervention likely to favour outcomes in the experimental intervention or the comparator
Free toothbrushes/toothpaste (quarterly)	No	Favour experimental
Referrals to social/food/housing services	No	Favour experimental

Risk of bias assessment

Responses underlined in green are potential markers for low risk of bias, and responses in red are potential markers for a risk of bias. Where questions relate only to sign posts to other questions, no formatting is used.

Signalling questions	Description	Response options
**Bias due to confounding**		
1.1 Is there potential for confounding of the effect of intervention in this study?<br>**If N/PN to 1.1:** the study can be considered to be at low risk of bias due to confounding and no further signalling questions need be considered	Single arm, no control group, 12-month follow-up with multiple unadjusted confounders (free supplies, co-interventions, child maturation).	Y
**If Y/PY to 1.1**: determine whether there is a need to assess time-varying confounding:		
1.2. Was the analysis based on splitting participants' follow up time according to intervention received?<br>**If N/PN**, answer questions relating to baseline confounding (1.4 to 1.6)<br>**If Y/PY**, go to question 1.3.	Simple pre/post comparison only, no time-split analysis.	N
1.3. Were intervention discontinuations or switches likely to be related to factors that are prognostic for the outcome?<br>**If N/PN**, answer questions relating to baseline confounding (1.4 to 1.6)<br>**If Y/PY**, answer questions relating to both baseline and time-varying confounding (1.7 and 1.8)	Single arm, no switching possible.	NA

**Questions relating to baseline confounding only**		
1.4. Did the authors use an appropriate analysis method that controlled for all the important confounding domains?	Paired pre/post GEE only; no adjustment for free supplies, co-interventions, or maturation	N
1.5. **If Y/PY to 1.4**: Were confounding domains that were controlled for measured validly and reliably by the variables available in this study?		NA
1.6. Did the authors control for any post-intervention variables that could have been affected by the intervention?	No post-intervention variables included in model	NA
**Questions relating to baseline and time-varying confounding**		
1.7. Did the authors use an appropriate analysis method that controlled for all the important confounding domains and for time-varying confounding?	No time-split analysis conducted	NA
1.8. **If Y/PY to 1.7**: Were confounding domains that were controlled for measured validly and reliably by the variables available in this study?		NA
**Risk of bias judgement**	Single-arm design, no control group, 12-month follow-up with unadjusted co-interventions (free supplies) and no time-varying analysis.	Serious
Optional: What is the predicted direction of bias due to confounding?		Favours experimental / Favours comparator / Unpredictable

Bias in selection of participants into the study		
2.1. Was selection of participants into the study (or into the analysis) based on participant characteristics observed after the start of intervention?<br>**If** N/PN **to 2.1:** go to 2.4	Eligibility based on baseline criteria only (age 24–72 months, diagnosed caries, no comorbidities)	N
2.2. **If Y/PY to 2.1**: Were the post-intervention variables that influenced selection likely to be associated with intervention?		NA
2.3 **If Y/PY to 2.2**: Were the post-intervention variables that influenced selection likely to be influenced by the outcome or a cause of the outcome?		NA
2.4. Do start of follow-up and start of intervention coincide for most participants?	Baseline visit = intervention start = pre-survey administration	Y
2.5. **If Y/PY to 2.2 and 2.3, or N/PN to 2.4**: Were adjustment techniques used that are likely to correct for the presence of selection biases?		NA
**Risk of bias judgement**	Eligibility criteria applied at baseline only, follow-up and intervention started simultaneously, and analysis included all participants with available data.	Low
Optional: What is the predicted direction of bias due to selection of participants into the study?		Favours experimental / Favours comparator / Towards null /Away from null / Unpredictable

Bias in classification of interventions		
3.1 Were intervention groups clearly defined?	MSB program well-described: app-guided, CHW-delivered, 3 visits, quarterly supplies	Y
3.2 Was the information used to define intervention groups recorded at the start of the intervention?	Baseline visit documented via MSB app	Y
3.3 Could classification of intervention status have been affected by knowledge of the outcome or risk of the outcome?	Single arm; all received MSB, no misclassification possible	N
**Risk of bias judgement**	Intervention clearly defined, consistently delivered, and documented via app.	Low
Optional: What is the predicted direction of bias due to classification of interventions?		Favours experimental / Favours comparator / Towards null /Away from null / Unpredictable

Bias due to deviations from intended interventions		
**If your aim for this study is to assess the effect of assignment to intervention, answer questions 4.1 and 4.2**		
4.1. Were there deviations from the intended intervention beyond what would be expected in usual practice?		PN
4.2. **If Y/PY to 4.1**: Were these deviations from intended intervention unbalanced between groups *and* likely to have affected the outcome?		NA
**If your aim for this study is to assess the effect of starting and adhering to intervention, answer questions 4.3 to 4.6**		
4.3. Were important co-interventions balanced across intervention groups?		NA
4.4. Was the intervention implemented successfully for most participants?		NA
4.5. Did study participants adhere to the assigned intervention regimen?		NA
4.6. **If N/PN to 4.3, 4.4 or 4.5**: Was an appropriate analysis used to estimate the effect of starting and adhering to the intervention?		NA
**Risk of bias judgement**	Pragmatic design explicitly allows real-world variation. High follow-up rate and app-guided delivery support fidelity.	Low
Optional: What is the predicted direction of bias due to deviations from the intended interventions?		Favours experimental / Favours comparator / Towards null /Away from null / Unpredictable

**Bias due to missing data**		
5.1 Were outcome data available for all, or nearly all, participants?	93.6% for primary outcome (therapeutic toothbrushing); but some secondary outcomes drop to n=572 (47%) with no explanation	PY
5.2 Were participants excluded due to missing data on intervention status?	Single arm, no intervention status to be missing	N
5.3 Were participants excluded due to missing data on other variables needed for the analysis?	Variable-level missingness substantial for some outcomes (sharing utensils, meal/snack location)	Y
5.4 **If PN/N to 5.1, or Y/PY to 5.2 or 5.3**: Are the proportion of participants and reasons for missing data similar across interventions?	Single arm; no breakdown of who had missing data or why	NI
5.5 **If PN/N to 5.1, or Y/PY to 5.2 or 5.3**: Is there evidence that results were robust to the presence of missing data?	No sensitivity analysis or imputation reported	PN
**Risk of bias judgement**	Primary outcome retention strong (93.6%), but substantial unexplained missingness for several secondary outcomes and no robustness checks.	Moderate
Optional: What is the predicted direction of bias due to missing data?		Favours experimental / Favours comparator / Towards null /Away from null / Unpredictable

**Bias in measurement of outcomes**		
6.1 Could the outcome measure have been influenced by knowledge of the intervention received?	Parent self-report; parents knew intervention goals, social desirability acknowledged by authors	Y
6.2 Were outcome assessors aware of the intervention received by study participants?	Same CHWs delivered intervention AND administered post-survey; no blinding possible	Y
6.3 Were the methods of outcome assessment comparable across intervention groups?	Single arm	N
6.4 Were any systematic errors in measurement of the outcome related to intervention received?	CHW rapport and prompting likely inflated positive responses despite mitigation attempts	Y
**Risk of bias judgement**	Parent self-report administered by the same CHWs who delivered the intervention creates strong social desirability bias. Authors acknowledge this explicitly but mitigation (verbal prompts) insufficient to eliminate it.	Serious
Optional: What is the predicted direction of bias due to measurement of outcomes?		Favours experimental

**Bias in selection of the reported result**		
Is the reported effect estimate likely to be selected, on the basis of the results, from...		
7.1. ... multiple outcome *measurements* within the outcome domain?		PN
7.2 ... multiple *analyses* of the intervention-outcome relationship?		PN
7.3 ... different *subgroups*?		PN
**Risk of bias judgement**	No registered protocol identified, but complete reporting of all outcomes including non-significant results reduces concern. Subgroup analyses appear pre-specified within the analysis plan.	Moderate
Optional: What is the predicted direction of bias due to selection of the reported result?		Favours experimental / Favours comparator / Towards null /Away from null / Unpredictable

Overall bias		
**Risk of bias judgement**	The single-arm design with no control group means changes cannot be attributed to the intervention alone, and parent-reported outcomes were administered by the same community health workers who delivered it, creating strong social desirability bias. Free oral hygiene supplies throughout the trial represent a major unadjusted co-intervention that may independently explain the toothbrushing improvements observed.	Serious
Optional: What is the overall predicted direction of bias for this outcome?		Favours experimental

This work is licensed under a Creative Commons Attribution-NonCommercial-NoDerivatives 4.0 International License.
