# Supplementary material for: Oral Health Interventions for Culturally and Linguistically Diverse Children and Caregivers: A RE‐AIM Systematic Review of Implementation Gaps
Source: Community Dent Oral Epidemiol. 2026 Jul 16;54(5):527–45. doi: 10.1111/cdoe.70090 (PMC13669799; doi:10.1111/cdoe.70090)
Supplement: Supplementary file 1 [file CDOE-54-527-s001.zip › cdoe70090-sup-0006-DataS2@Supplementary_File_S2_Search_Strategies.docx]

| Supplementary Data 2. Electronic databases searched and search strategies used in the systematic review (from 2010 up to Dec 2025) | | |
| --- | --- | --- |
| **Database** | **Search strategy used** | **Hits** |
| Web of Science  (https://www.webofscience.com/wos/woscc/smart-search) | "Culturally Competent Care" OR "Diversity, Equity and Inclusion" OR "Cultural Diversity" OR "Transients and Migrants" OR "Culturally and Linguistically Diverse" OR CALD OR "culturally diverse" OR "linguistically diverse" OR Transients OR migrant* OR Emigrants OR Immigrant* OR refugee OR "ethnic minority" OR "Ethnic Groups" OR Acculturation OR "Cross-Cultural Comparison" AND Adolescent OR Infant OR Child OR Child* OR kid OR kids OR adolescent OR adolescen* OR Pediatric OR Paediatric OR youth* OR "young people" OR teen* OR Parent* OR Carer* OR caregiver* OR caregivers  AND "Oral Health" OR "Dental Care" OR "Dental Caries" OR "oral health" OR "dental service*" OR dentist OR "dental hygiene" OR "Dental Caries" OR toothache OR "dental plaque" OR "oral care" OR "oral hygiene" OR "caries prevention" OR (("access to care" OR "health disparities") AND oral OR "oral health literacy" | 1014 |
| Pubmed/MEDLINE  (https://pubmed.ncbi.nlm.nih.gov/?otool=iauqulib) | "Culturally Competent Care"[Mesh] OR "Diversity, Equity, Inclusion"[Mesh] OR "Cultural Diversity"[Mesh] OR "Transients and Migrants"[Mesh] OR "Culturally and Linguistically Diverse"[tiab] OR CALD[tiab] OR "culturally diverse"[tiab] OR "linguistically diverse"[tiab] OR Transients[tiab] OR migrant*[tiab] OR Emigrants[tiab] OR Immigrant*[tiab] OR refugee[tiab] OR ethnic minority[tiab] OR Ethnic Groups[tiab] OR Acculturation[tiab] OR Cross-Cultural Comparison[tiab]  AND "Adolescent"[Mesh] OR "Infant"[Mesh] OR Child[mh] OR Child*[tiab] OR kid[tiab] OR kids[tiab] OR adolescent[tiab] OR adolescen*[tiab] OR Pediatric[tiab] OR Paediatric[tiab] OR youth*[tiab] OR "young people"[tiab] OR teen*[tiab] OR Parent*[tiab] OR Carer*[tiab] OR caregiver*[tiab] OR caregivers[tiab] AND "Oral Health"[Mesh] OR "Dental Care"[Mesh] OR “Dental Caries"[Mesh] OR oral health[tiab] OR dental service*[tiab] OR dentist[tiab] OR dental hygiene[tiab] OR Dental Caries[tiab] OR toothache[tiab] OR dental plaque[tiab] OR oral care[tiab] OR oral hygiene[tiab] OR "caries prevention"[tiab] OR (("access to care"[tiab] OR "health disparities"[tiab]) AND oral[tiab]) OR "oral health literacy"[tiab] | 771 |
| Scopus  (https://www.scopus.com) | ( ( ( ( "oral health" OR "dental services" OR dentist OR "dental hygiene" OR "Dental Caries" OR toothache OR "dental plaque" ) AND ( "Culturally Competent Care" OR "Diversity, Equity, Inclusion" OR "Cultural Diversity" OR "Culturally and Linguistically Diverse" OR cald OR "culturally diverse" OR "linguistically diverse" OR transient OR migrant* OR emigrants OR immigrant* OR refugee OR "ethnic minority" OR "Ethnic Groups" OR acculturation OR "Cross-Cultural Comparison" ) ) AND ( child OR child* OR kid OR kids OR adolescent OR adolescen* OR pediatric OR paediatric OR youth* OR "young people" OR teen* OR parent* OR carer* OR caregiver* OR caregivers ) ) AND ( barrier* OR obstacle* OR enabl* OR facilitat* OR inequality OR inequalit* ) ) AND ( "delivery of health care" OR "delivery of health care" OR "Health Services Accessibility" OR "health care" OR "oral care" OR "dental care" OR "health service*" OR "oral service*" ) AND ( LIMIT-TO ( SUBJAREA , "DENT" ) ) AND ( LIMIT-TO ( DOCTYPE , "ar" ) OR LIMIT-TO ( DOCTYPE , "re" ) ) | 749 |
| Embase  (https://www.embase.com/search/) | 'transcultural care'/exp OR 'diversity, equity and inclusion'/exp OR 'cultural diversity'/exp OR 'Culturally and Linguistically Diverse':ti,ab OR CALD:ti,ab OR 'culturally diverse':ti,ab OR 'linguistically diverse':ti,ab OR Transients:ti,ab OR migrant*:ti,ab OR Emigrants:ti,ab OR Immigrant*:ti,ab OR refugee:ti,ab OR 'ethnic minority':ti,ab OR 'Ethnic Groups':ti,ab OR Acculturation:ti,ab OR 'Cross-Cultural Comparison':ti,ab AND'adolescent'/exp OR 'child'/exp OR 'infant'/exp OR Child*:ti,ab OR kid:ti,ab OR kids:ti,ab OR adolescent:ti,ab OR adolescen*:ti,ab OR Pediatric:ti,ab OR Paediatric:ti,ab OR youth*:ti,ab OR 'young people':ti,ab OR teen*:ti,ab OR Parent*:ti,ab OR Carer*:ti,ab OR caregiver*:ti,ab OR caregivers:ti,ab AND 'dental health'/exp OR 'dental caries'/exp OR 'oral health':ti,ab OR “dental care”:ti,ab OR 'dental service*':ti,ab OR dentist:ti,ab OR 'dental hygiene':ti,ab OR 'Dental Caries':ti,ab OR toothache:ti,ab OR 'dental plaque':ti,ab OR 'oral care':ti,ab OR 'oral hygiene':ti,ab OR 'caries prevention':ti,ab OR 'access to care':ti,ab OR 'health disparities':ti,ab AND oral:ti,ab OR 'oral health literacy':ti,ab AND [embase]/lim AND ([article]/lim OR [article in press]/lim OR [review]/lim) | 567 |
| **CINAHL Complete (EBSCOhost)**  (https://research.ebsco.com/c/nsk4sj/search/advanced/filters?autocorrect=y&defaultdb=ccm) | (MH "Culturally Competent Care+") OR (MH "Diversity, Equity, Inclusion+") OR (MH "Cultural Diversity+") OR (MH "Transients and Migrants+") OR (TI "Culturally and Linguistically Diverse" OR AB "Culturally and Linguistically Diverse") OR (TI CALD OR AB CALD) OR (TI "culturally diverse" OR AB "culturally diverse") OR (TI "linguistically diverse" OR AB "linguistically diverse") OR (TI Transients OR AB Transients) OR (TI migrant* OR AB migrant*) OR (TI Emigrants OR AB Emigrants) OR (TI Immigrant* OR AB Immigrant*) OR (TI refugee OR AB refugee) OR (TI "ethnic minority" OR AB "ethnic minority") OR (TI "Ethnic Groups" OR AB "Ethnic Groups") OR (TI Acculturation OR AB Acculturation) OR (TI "Cross-Cultural Comparison" OR AB "Cross-Cultural Comparison") AND (MH Adolescent+) OR (MH Infant+) OR (MH Child+) OR (TI Child* OR AB Child*) OR (TI kid OR AB kid) OR (TI kids OR AB kids) OR (TI adolescent OR AB adolescent) OR (TI adolescen* OR AB adolescen*) OR (TI Pediatric OR AB Pediatric) OR (TI Paediatric OR AB Paediatric) OR (TI youth* OR AB youth*) OR (TI "young people" OR AB "young people") OR (TI teen* OR AB teen*) OR (TI Parent* OR AB Parent*) OR (TI Carer* OR AB Carer*) OR (TI caregiver* OR AB caregiver*) OR (TI caregivers OR AB caregivers)  AND (MH "Oral Health+") OR (MH "Dental Care+") OR (MH "Dental Caries+") OR (TI "oral health" OR AB "oral health") OR (TI "dental service*" OR AB "dental service*") OR (TI dentist OR AB dentist) OR (TI "dental hygiene" OR AB "dental hygiene") OR (TI "Dental Caries" OR AB "Dental Caries") OR (TI toothache OR AB toothache) OR (TI "dental plaque" OR AB "dental plaque") OR (TI "oral care" OR AB "oral care") OR (TI "oral hygiene" OR AB "oral hygiene") OR (TI "caries prevention" OR AB "caries prevention") OR (((TI "access to care" OR AB "access to care") OR (TI "health disparities" OR AB "health disparities")) AND (TI oral OR AB oral)) OR (TI "oral health literacy" OR AB "oral health literacy") | 340 |
| Cochrane Library  (https://www.cochranelibrary.com/) | [mh "Culturally Competent Care"] OR [mh "Diversity, Equity, Inclusion"] OR [mh "Cultural Diversity"] OR [mh "Transients and Migrants"] OR "Culturally and Linguistically Diverse":ti,ab OR CALD:ti,ab OR "culturally diverse":ti,ab OR "linguistically diverse":ti,ab OR Transients:ti,ab OR migrant*:ti,ab OR Emigrants:ti,ab OR Immigrant*:ti,ab OR refugee:ti,ab OR "ethnic minority":ti,ab OR "Ethnic Groups":ti,ab OR Acculturation:ti,ab OR "Cross-Cultural Comparison":ti,ab AND [mh Adolescent] OR [mh Infant] OR [mh Child] OR Child*:ti,ab OR kid:ti,ab OR kids:ti,ab OR adolescent:ti,ab OR adolescen*:ti,ab OR Pediatric:ti,ab OR Paediatric:ti,ab OR youth*:ti,ab OR "young people":ti,ab OR teen*:ti,ab OR Parent*:ti,ab OR Carer*:ti,ab OR caregiver*:ti,ab OR caregivers:ti,ab AND [mh "Oral Health"] OR [mh "Dental Care"] OR [mh "Dental Caries"] OR "oral health":ti,ab OR ("dental" NEXT service*):ti,ab OR dentist:ti,ab OR "dental hygiene":ti,ab OR "Dental Caries":ti,ab OR toothache:ti,ab OR "dental plaque":ti,ab OR "oral care":ti,ab OR "oral hygiene":ti,ab OR "caries prevention":ti,ab OR (("access to care":ti,ab OR "health disparities":ti,ab)) AND oral:ti,ab) OR "oral health literacy":ti,ab | 307 |
| Total |  | 3748 |
